# Genome-wide linkage disequilibrium and genetic diversity in five populations of Australian domestic sheep

**DOI:** 10.1186/s12711-015-0169-6

**Published:** 2015-11-24

**Authors:** Hawlader Abdullah Al-Mamun, Samuel A Clark, Paul Kwan, Cedric Gondro

**Affiliations:** School of Environmental and Rural Science, University of New England, Armidale, NSW 2351 Australia; School of Science and Technology, University of New England, Armidale, NSW 2351 Australia

## Abstract

**Background:**

Knowledge of the genetic structure and overall diversity of livestock species is important to maximise the potential of genome-wide association studies and genomic prediction. Commonly used measures such as linkage disequilibrium (LD), effective population size (*N*_*e*_), heterozygosity, fixation index (*F*_ST_) and runs of homozygosity (ROH) are widely used and help to improve our knowledge about genetic diversity in animal populations. The development of high-density single nucleotide polymorphism (SNP) arrays and the subsequent genotyping of large numbers of animals have greatly increased the accuracy of these population-based estimates.

**Methods:**

In this study, we used the Illumina OvineSNP50 BeadChip array to estimate and compare LD (measured by *r*^*2*^ and *D*′), *N*_*e*_, heterozygosity, *F*_ST_ and ROH in five Australian sheep populations: three pure breeds, i.e., Merino (MER), Border Leicester (BL), Poll Dorset (PD) and two crossbred populations i.e. F1 crosses of Merino and Border Leicester (MxB) and MxB crossed to Poll Dorset (MxBxP).

**Results:**

Compared to other livestock species, the sheep populations that were analysed in this study had low levels of LD and high levels of genetic diversity. The rate of LD decay was greater in Merino than in the other pure breeds. Over short distances (<10 kb), the levels of LD were higher in BL and PD than in MER. Similarly, BL and PD had comparatively smaller *N*_*e*_ than MER. Observed heterozygosity in the pure breeds ranged from 0.3 in BL to 0.38 in MER. Genetic distances between breeds were modest compared to other livestock species (highest *F*_ST_ = 0.063) but the genetic diversity within breeds was high. Based on ROH, two chromosomal regions showed evidence of strong recent selection.

**Conclusions:**

This study shows that there is a large range of genome diversity in Australian sheep breeds, especially in Merino sheep. The observed range of diversity will influence the design of genome-wide association studies and the results that can be obtained from them. This knowledge will also be useful to design reference populations for genomic prediction of breeding values in sheep.

**Electronic supplementary material:**

The online version of this article (doi:10.1186/s12711-015-0169-6) contains supplementary material, which is available to authorized users.

## Background

The process of sheep domestication began between 9000 and 11,000 years ago. Over thousands of years, humans have selected sheep for different desirable production traits such as wool, milk and meat. This artificial selection combined with natural adaptation to new environments as sheep were introduced throughout the world has led to a broad spectrum of phenotypic diversity with more than one thousand different sheep breeds [1]. Australia is one of the world’s largest sheep producers for wool and meat, with the Merino breed being the most economically important. Merino animals account for approximately 75 % of all Australian sheep (Year book Australia 2003, Australian Bureau of Statistics) and another 12 % of the population are Merino x Border Leicester F1 crosses that produce high-quality females used for meat production. These animals are commonly crossed with meat breeds such as Poll Dorset for the production of prime lambs (http://www.polldorset.org.au). The three pure breeds Merino, Poll Dorset, Border Leicester and their crosses (Merino × Border Leicester and Merino × Border Leicester × Poll Dorset) represent more than 90 % of the Australian sheep population.

Understanding the genetic diversity of these key sheep breeds in Australia is important to design and interpret studies that investigate genome-wide association and genomic prediction. For example, the amount of diversity in a population is a key indicator of the amount of phenotypic data (reference data) that is required to obtain accurate genomic predictions. This is also important when interpreting genome-wide association studies (GWAS) data since high levels of diversity reduce the likelihood that highly significant markers are at a large distance from the quantitative trait locus (QTL) that underlies variation in phenotype and allows for easier identification of possible functional regions.

Genetic diversity can be estimated from pedigree data or from molecular marker data. Estimates of genetic diversity are more robust when marker data is used and particularly so when pedigree records are poor or shallow. However, this advantage is small or absent when low-density markers such as microsatellites are used [2–4]. The high-density single nucleotide polymorphism (SNP) arrays that are currently available have provided the opportunity to estimate genetic diversity parameters in livestock at a much higher level of definition than was previously possible.

There are a number of methods that can be used to estimate genetic diversity using marker data. These include observed and expected heterozygosity [5, 6], runs of homozygosity (ROH) [7], Wright’s *F* statistic (*F*_ST_) [8], linkage disequilibrium (LD) and effective population size (*N*_*e*_) [9].

Heterozygosity measures the genetic variation within a population and is one of the most widely used genetic diversity parameters [10]. A high level of heterozygosity indicates more genetic variability while a low level indicates little genetic variability and a small *N*_*e*_. Wright’s *F* statistics (*F*_IT_, *F*_IS_, *F*_ST_) are widely used to estimate genetic diversity within and between populations [3, 11]. Runs of homozygosity are contiguous stretches of homozygous genotypes (e.g., an individual inherits the same haplotype from both parents). Long ROH could be a sign of recent inbreeding in a population whereas shorter ROH suggest loss of genetic diversity either from a population bottleneck or a founder effect (e.g., breed formation in livestock).

LD between any two markers reflects the extent of non-random association between them. LD underpins selection decisions in a wide range of livestock species that have adopted genetic technologies for selection purposes. Marker-assisted selection (MAS), genomic selection and GWAS all largely depend on the extent of LD within a population. It is the extent of LD that determines the minimum number of markers required for a successful genome-wide study; with LD remaining high over longer chromosomal segments, fewer markers are needed. Conversely, denser panels are required if LD decays rapidly. The pattern of LD decay also provides information on the evolutionary history of a population and can be used to estimate, e.g., the ancestral *N*_*e*_ [12, 13]. *N*_*e*_ size and other genetic events such as selection, migration, mutation and recombination events influence the extent of LD within a population [14]. Comparison of the extent of LD between breeds is therefore informative about the overall diversity level in a species and can help us understand the patterns of selection that individual breeds have been subjected to. Due to its importance, various studies have reported LD estimates in various livestock species, e.g. cattle [15–17], pig [18], horse [19], chicken [20] and sheep [21–23].

Our objective was to describe and compare LD patterns, and the level and structure of genetic diversity in the five commercial Australian sheep populations mentioned above. The results are expected to provide valuable information for the design and analysis of genetic association studies and genomic selection, as well as for the management of genetic resources in the most economically important Australian sheep populations.

## Methods

### Ethics statement

Samples for genotyping were collected under approval number 344 AEC12-049 of the University of New England Animal Ethics Committee.

### Animal resources

The study consisted of 1273 sheep chosen from the Australian Sheep CRC Information Nucleus flock from five different populations: three pure breeds i.e., Border Leicester (BL; *n* = 253), Merino (MER; *n* = 265), Poll Dorset (PD; *n* = 264), and two crossbred populations i.e., Merino and Border Leicester F1 crosses (MxB; *n* = 260), and crosses of Merino × Border Leicester with Poll Dorset (MxBxP; *n* = 231). MER is a wool breed, while BL and PD are primarily meat breeds; each breed was separately selected for its own specific purposes. MxB and MxBxP are straight F1 crosses from the pure breeds rather than terminal composite breeds and are not subjected to artificial selection. These crosses are often used for both wool and meat production. All animals were genotyped using the Illumina OvineSNP50 BeadChip (Illumina Inc., San Diego, CA, USA), which includes 54,241 SNPs.

### Genotyping and quality control

A number of quality control measures were applied to all SNPs as follows: SNPs were removed if they had a call rate <95 %, a GC score <0.6, a minor allele frequency (MAF) <0.01 and if deviation in SNP heterozygosity was greater than three standard deviations from the mean. Data was also removed if SNP genotypes deviated from the Hardy-Weinberg equilibrium (for a P value cut-off of 1 × 10^−15^) and had no assigned genomic location. Markers on sex chromosomes were also excluded from the analysis. Quality control was performed using snpQC [24]. Missing genotypes were imputed using BEAGLE 3 [25].

### SNP genome coordinates on the ovine genome sequence assembly

Chromosomal coordinates for each SNP were obtained by aligning the region that covered approximately 120 bp around each SNP, to the latest release of the ovine genome sequence assembly, Oar_v3.1, by BLAST (http://blast.ncbi.nlm.nih.gov/Blast.cgi). Forty-one markers were excluded from the analysis because they had no assigned genomic location in Oar_v3.1.

### Genetic analysis of gene diversity

Gene diversity or expected heterozygosity (*H*_*E*_) was calculated as a measure of genetic diversity. *H*_*E*_ for each SNP was calculated separately and then averaged to find the average genetic diversity for each breed. Population relatedness was evaluated using pair-wise estimates of *F*_ST_. First, expected heterozygosities for subpopulations (*HS*) and for the total population (*HT*) were calculated for each locus for each pair of populations, then *HS* and *HT* were averaged across all loci. *H* values (expected heterozygosities for populations and combined populations) were calculated as 1 minus the sum of the squared allele frequencies at a locus. To calculate *HT*, allele frequencies between two populations were averaged before the calculation. To calculate *HS*, *H* values were averaged after the calculation. In both cases, the averages were weighted by the relative sizes of the two populations. *F*_*ST*_ was then calculated for each pair of populations as (*HT* − *HS*)/*HT*. A principal component analysis (PCA) was used to examine the genetic structure of the five populations. The PCA was performed using a genomic relationship matrix [26] to define the covariance between animals. The relationship between the first two principal components was examined to show the relationship between the five populations.

### Measure of runs of homozygosity

Runs of homozygosity were defined for individuals in each of the five populations using PLINK v1.07 [27] with sliding windows of 1000 kb across the genome to estimate homozygosity. A maximum of two SNPs with missing genotypes were allowed per window and up to one possible heterozygous genotype was permitted. To minimize the number of false positives, the minimum number of SNPs that constituted a ROH (*l*) was calculated by a method similar to that proposed by [28]:

1 $$l = \frac{{\log_{e} \frac{\alpha }{{n_{s} \cdot n_{i} }}}}{{\log_{e} (1 - het)}} ,$$

where *n*_*s*_ was the number of genotyped SNPs per individual, *n*_*i*_ was the number of individuals, *α* was the percentage of false positive ROH (set to 0.05 in the present study) and *het* was the mean SNP heterozygosity across all SNPs. Since very short and common ROH are often due to LD, ROH that were <500 kb long were removed. Finally, the maximum gap between consecutive homozygous SNPs was set to 250 kb.

Runs of homozygosity were estimated for each individual and then categorized based on ROH length (1–5 Mb, 5–10 Mb, 10–15 Mb, 15–20 Mb, 20–25 Mb, and >25 Mb). The mean sum of ROH within each ROH category was calculated by adding up the length of all ROH for each individual in each ROH category and then the results were averaged per breed population. The percentage of occurrences of a SNP in a ROH was calculated for each SNP by counting the number of times the SNP was detected in a ROH across the dataset of the whole population.

### Linkage disequilibrium and haplotype blocks

As a measurement of LD, we used the two most commonly used statistics, *D*′ and *r*^*2*^, for an easy comparison of our results with those of other reports. Haploview v4.2 [29] was used to estimate LD. For each breed, each chromosome was analysed and all pairwise LD combinations (*D*′ and *r*^*2*^) were estimated. SNP pairs were grouped according to their pairwise distance into 14 categories: <10 kb, 10–20 kb, 20–40 kb, 40–60 kb, 60–100 kb, 100–200 kb, 200–500 kb, 500 kb–1 Mb, 1–2 Mb, 2–5 Mb, 5–10 Mb, 10–20 Mb, 20–50 Mb, and >50 Mb. Average LD within each group was calculated for each breed. Average LD with neighbouring pairs of SNPs and average LD across the chromosome were also estimated for each chromosome. For non-syntenic SNPs, a subset of SNPs from the whole genome was used to estimate LD. For each autosome, a random representative sample of SNPs was selected to obtain an estimate of LD (5 % of the SNPs for each autosome). Haploview v4.2 was also used to identify the haplotype blocks present in each chromosome. Haplotype blocks were defined using the method described in [30]. Two SNPs were considered to be in strong LD if the upper one-sided 95 % confidence bound of *D*′ was higher than 0.98 and if the lower bound was higher than 0.7.

### Effective population size and inbreeding coefficients

Effective population size (*N*_*e*_) was calculated for each breed using the default parameters and the random mating model in the software NEESTIMATOR v2 [31]. A bias-corrected version of the LD method [32] was used to obtain the final estimate of *N*_*e*_.

Marker-based inbreeding coefficients for each breed were estimated using the GCTA software [33]. Individual allele frequencies for each population were calculated and three different metrics for *F* values were calculated by GCTA: *F*_*1*_ based on the variance of the additive genotype; *F*_*2*_ based on the excess of homozygotes; and *F*_*3*_ based on the correlation between uniting gametes [33]. Inbreeding coefficients for each breed were calculated by averaging inbreeding coefficients of all individuals for that breed.

## Results

### Descriptive statistics

From the initial set of 54,241 SNPs, 1450 (2.69 %) non-autosomal SNPs and 314 SNPs that had no chromosome assignment were removed. Another 3837 SNPs were excluded: 1662 because minor allele frequency (MAF) was <0.01, 1838 because SNP call rate (CR_SNP_) was <0.95, and 337 because they deviated from the HWE. Forty-one SNPs could not be mapped to the ovine Genome Assembly v3.1 and were removed from the analysis. No individual was removed due to low call rates (CR_IND_). A total of 48,599 SNPs across the five populations met the filtering criteria and were included in the final analysis. The distributions of the SNPs after filtering and the average distances between adjacent SNPs on each chromosome are in Additional file 1: Table S1. SNPs were uniformly distributed across all autosomes since marker density was similar for all chromosomes with the average distance between SNPs ranging from 47.26 kb on OAR8 (OAR for *Ovis aries* chromosome) to 61.62 kb on OAR24. Autosomes varied in size, with OAR24 being the shortest (42.03 Mb) and OAR1 the longest chromosome (275.61 Mb). After filtering, the number of SNPs on each chromosome ranged from 683 on OAR24 to 5484 on OAR1. The distribution of MAF differed between populations (see Additional file 1: Table S1; Additional file 2: Figure S1). Additional file 2: Figure S1 shows that BL and PD had an excess of SNPs with a low MAF (<0.1) compared to MER and the two crossbred populations.

### Genetic diversity and population structure

**Fig. 1:**
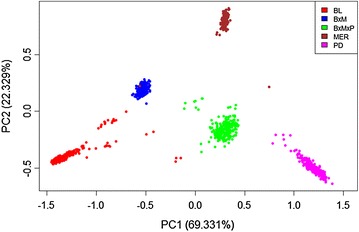
PCA plot of the genetic relationship matrix for the five populations studied. The three pure breeds were clearly separated and the first two principal components explained about 91.7 % of the total variation

Analysis of genetic diversity using the average observed heterozygosity and average expected heterozygosity (Table 1) showed that genetic diversity was lowest for BL, with *H*_*e*_ and *H*_*o*_ estimates both equal to 0.30, followed by PD, with *H*_*e*_ and *H*_*o*_ estimates both equal to 0.34. Among the pure breeds, MER was the most diverse with *H*_*e*_ and *H*_*o*_ estimates both equal to 0.38. Compared to the pure breeds, the crosses had a higher level of genetic diversity. We also investigated the level of relatedness between populations by calculating the pairwise *F*_ST_ (Table 1). The two most distantly related breeds are BL and MER with an average pairwise *F*_ST_ value of 0.062. The smallest average pairwise *F*_ST_ was observed between the two crosses (*F*_ST_ = 0.013), which were the most closely related pair. These estimates reflected expectations since MER and BL contributed to both crosses. Higher average *F*_ST_ values were observed for every pair-wise comparison of populations involving MER. Figure 1 shows the relationship between the first two principal components, which explained 91.7 % of the total variation and separated the populations into their respective breed groups.

**Table 1:** Genetic diversity within the five sheep populations studied

	*F*_ST_				% of polymorphic SNPs	*H*_*e*_	*H*_*o*_
	PD	MER	BxM	BxMxP			
BL	0.053	0.062	0.019	0.033	99.21	0.30	0.30
PD		0.053	0.036	0.016	98.74	0.34	0.34
MER			0.045	0.043	99.86	0.38	0.38
BxM				0.013	99.89	0.37	0.403
BxMxP					99.96	0.38	0.404

*H*_*e*_ expected heterozygosity

*H*_*o*_ observed heterozygosity

For each SNP and for each breed, *H*_*e*_, *Ho,* and *F*_*ST*_ were estimated and averaged

### Runs of homozygosity

**Fig. 2:**
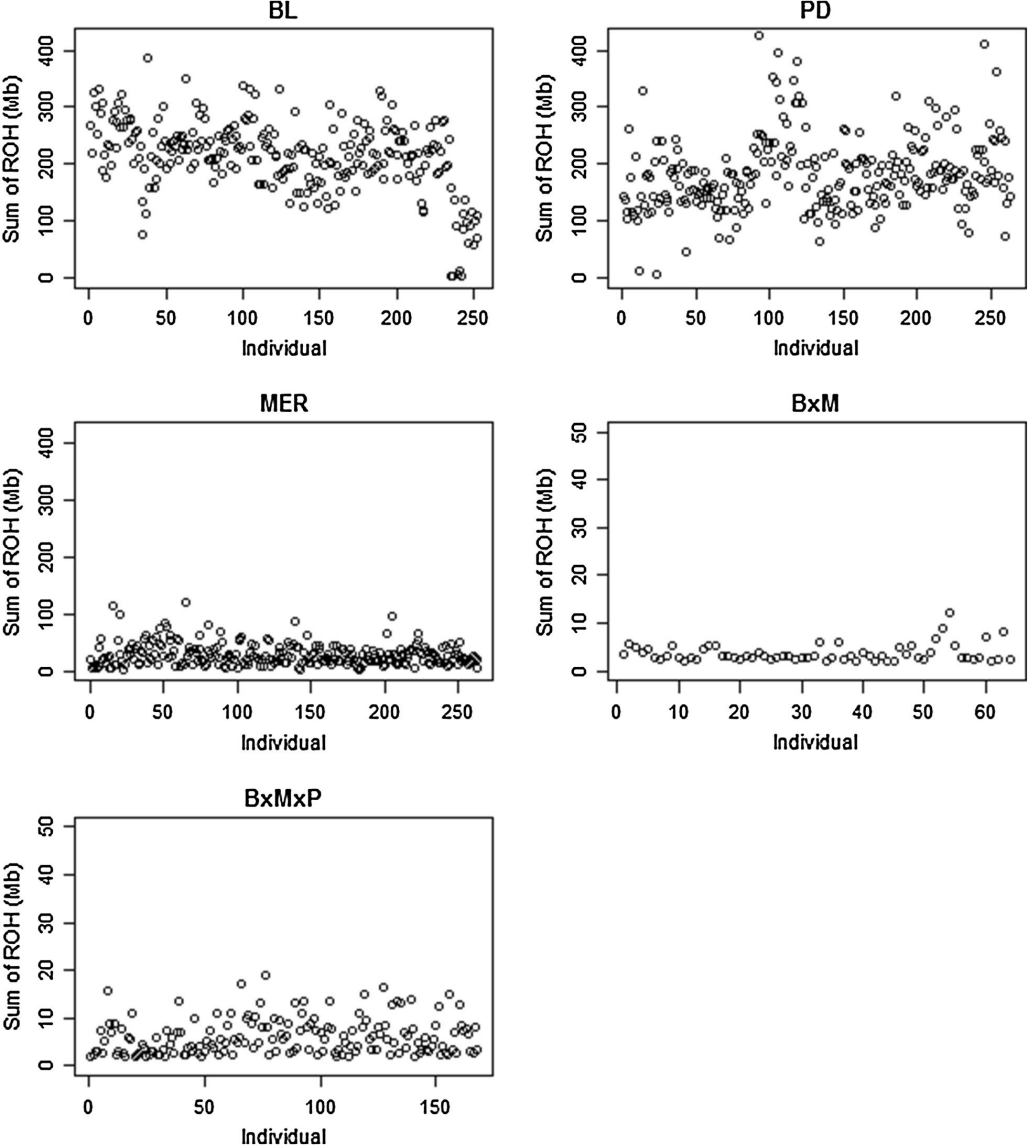
Sum of total runs of homozygosity (ROH) per animal for each population. Among the pure breeds, sums of ROH per animal were larger for BL and PD than for MER. Note the difference in scales used for the pure breeds and the crossbred populations

The number of ROH differed significantly between populations. BL had the largest total number of ROH (12,561) followed by PD (9875) and MER (2008). There were 49.65, 37.89 and 7.57 ROH per animal for the BL, PD and MER breeds, respectively. As expected, we found that the pure breeds had more ROH across the whole genome than the crosses. In fact, MxB and MxBxP had, in total, only 80 and 331 ROH. Figure 2 shows the sum of ROH (in Mb) per animal genome for each of the five populations. There were clear differences in both the levels and variation of ROH frequency. BL and PD had a larger sum of ROH per animal genome than MER and the crossbred populations.

**Fig. 3:**
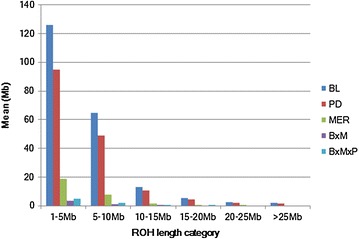
Average ROH per population. The average length of ROH (in Mb) for each population across six interval sizes. For each animal, within each ROH length category, ROH were summed up and then averaged per population

Among the 1273 animals, 1015 (80 %) had at least one ROH longer than 1 Mb and 751 (59 %) had at least one ROH longer than 5 Mb. If we consider only the pure breeds, then all animals had at least one ROH longer than 1 Mb and 688 of the 782 animals (88 %) had at least one ROH longer than 5 Mb. There are clear differences between breeds in the frequencies of the ROH of various lengths (Fig. 3). Two breeds, BL and PD, had on average, a larger part of their genome that contained ROH of 1 to 5 Mb (BL = 126.06 Mb and PD = 94.88 Mb). In all five populations, most of the ROH were shorter and ranged from 1 to 10 Mb (Fig. 3). No crossbred animal had a ROH longer than 20 Mb. In our study, the three animals with the highest level of homozygosity were PD individuals with 427.2, 410.5 and 396.45 Mb of their genome classified as ROH (data not shown), which is close to 20 % of the genome.

**Fig. 4:**
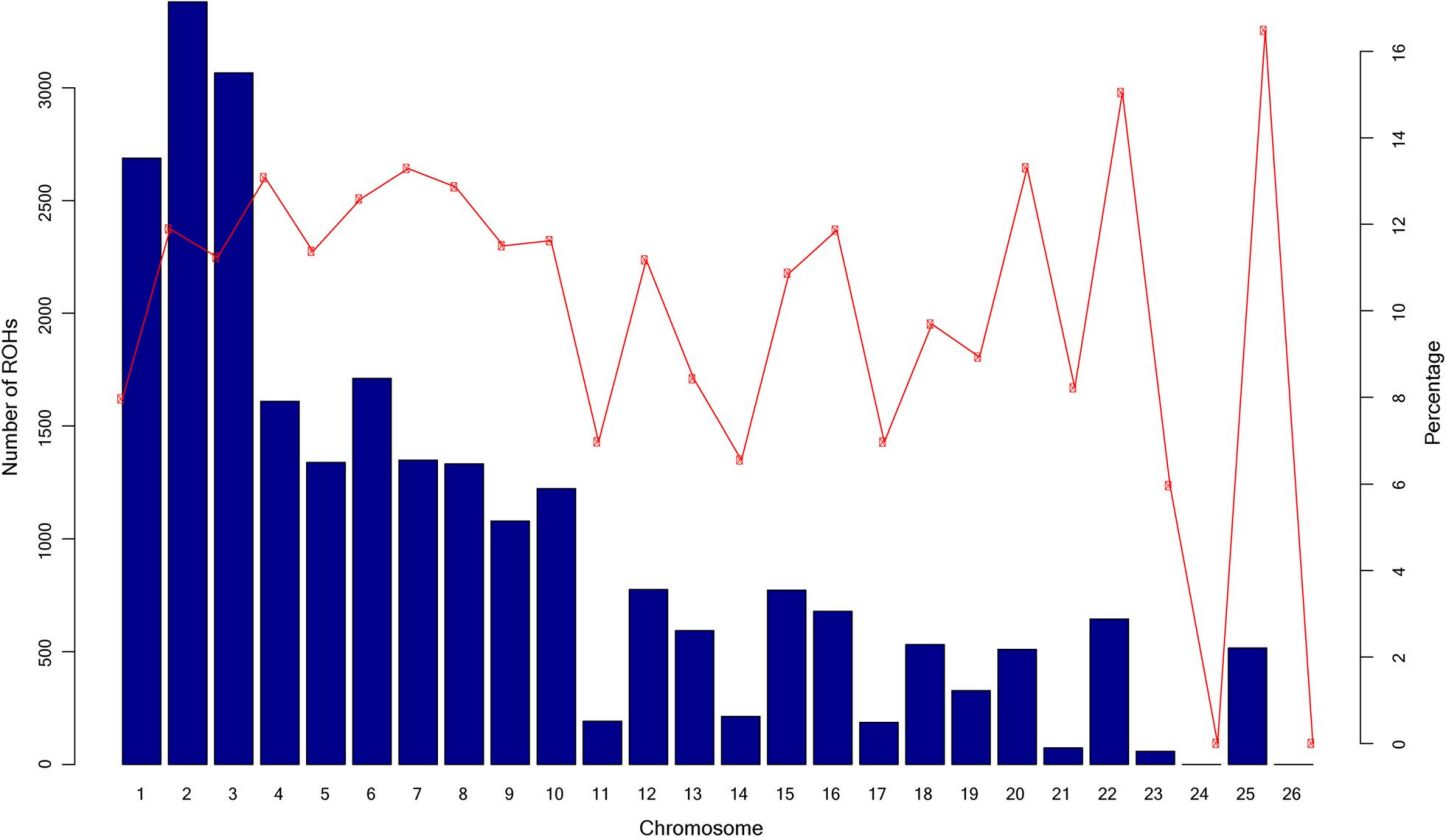
Number of ROH per chromosome and percentage of coverage per chromosome. The *bars* show the total number of ROH per chromosome identified in the 1015 animals that had at least one ROH. The *line* shows the average percentage of ROH for each chromosome. To calculate the percentage of ROH per chromosome, the mean ROH length was calculated by summing all ROH (in Mb) on a chromosome and then dividing by the number of animals that had ROH on that chromosome. The mean ROH length was then divided by the chromosome length (in Mb) and converted to percentage

**Fig. 5:**
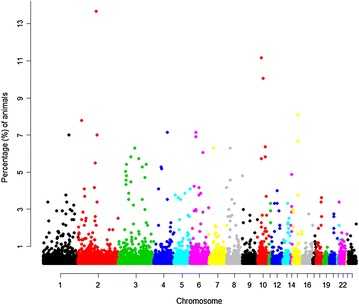
Incidence of each SNP in a ROH. Note that the last chromosome included in this figure is OAR25 because no ROH were identified on OAR24 and 26

OAR2 had the largest number of ROH (3381 for 543 animals) with on average 11.89 % of the chromosome consisting of ROH. Generally, the number of ROH per chromosome tended to decrease with chromosome length (Fig. 4). The largest proportion of the genome in ROH was observed for OAR25 and OAR22 with 16.48 and 15.05 %, respectively. SNP OAR2_119604666.1 on OAR2 was the most frequently found in ROH (174 occurrences); followed by OAR10_26604546.1 and s35658.1 (both on OAR10) with 142 and 128 occurrences. We also investigated LD between SNPs that were located in the vicinity of these three SNPs using Haploview [29] and found that most SNPs in these regions were in high LD with each other. In addition, OAR4, 6 and 15 contained SNPs that occurred at high frequencies in ROH (Fig. 5). In this data, no ROH were found on OAR24 and 26.

### Linkage disequilibrium analysis

**Fig. 6:**
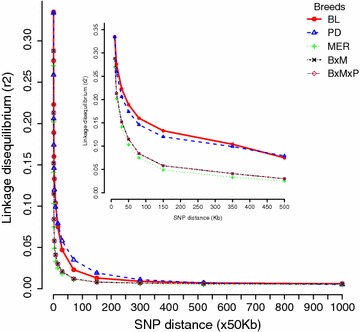
Average *r* ^*2*^ values for each population

Estimates of LD based on *r*^2^ values were different for each of the five populations (Fig. 6). Short-range LD was observed in all populations but the rate of LD decay differed between populations. LD was present over larger distances in the BL and PD populations but dropped quickly with increasing distance in the MER and the two crossbred populations. For SNPs up to 10 kb apart, the average *r*^*2*^ values were equal to 0.34 (BL), 0.27 (MER), 0.33 (PD), 0.29 (MxB), and 0.3 (MxBxP). Average LD between adjacent SNPs per chromosome was also estimated and some variation in the extent of LD on different chromosomes was observed for the five populations. In each population, the maximum average LD between adjacent SNPs was observed on OAR10 and was equal to 0.25 (BL), 0.15 (MER), 0.21 (PD), 0.17 (MxB), and 0.16 (MxBxP). However, the minimum average LD between adjacent SNPs was found for different chromosomes in the five populations (see Additional file 1: Table S2).

Linkage disequilibrium for each population was also estimated for all possible pairs of SNPs on the autosomes using *D*′. Similarly to the *r*^*2*^ values, *D*′ also decreased as pairwise marker distances increased; however, the rate of decay was less pronounced for *D*′ than for *r*^2^ (see Additional file 2: Figure S2). More details about LD analysis using D′ are included in Additional file 1: Tables S3 and S4 and Additional file 2: Figure S2.

Average *r*^*2*^ and *D*′ values for all possible pairs of SNPs on each chromosome were calculated and an inverse relationship between chromosome length and average *D*′ was observed for each population (see Additional file 1: Tables S4, S5).

### Pattern of haplotype blocks by breed

**Fig. 7:**
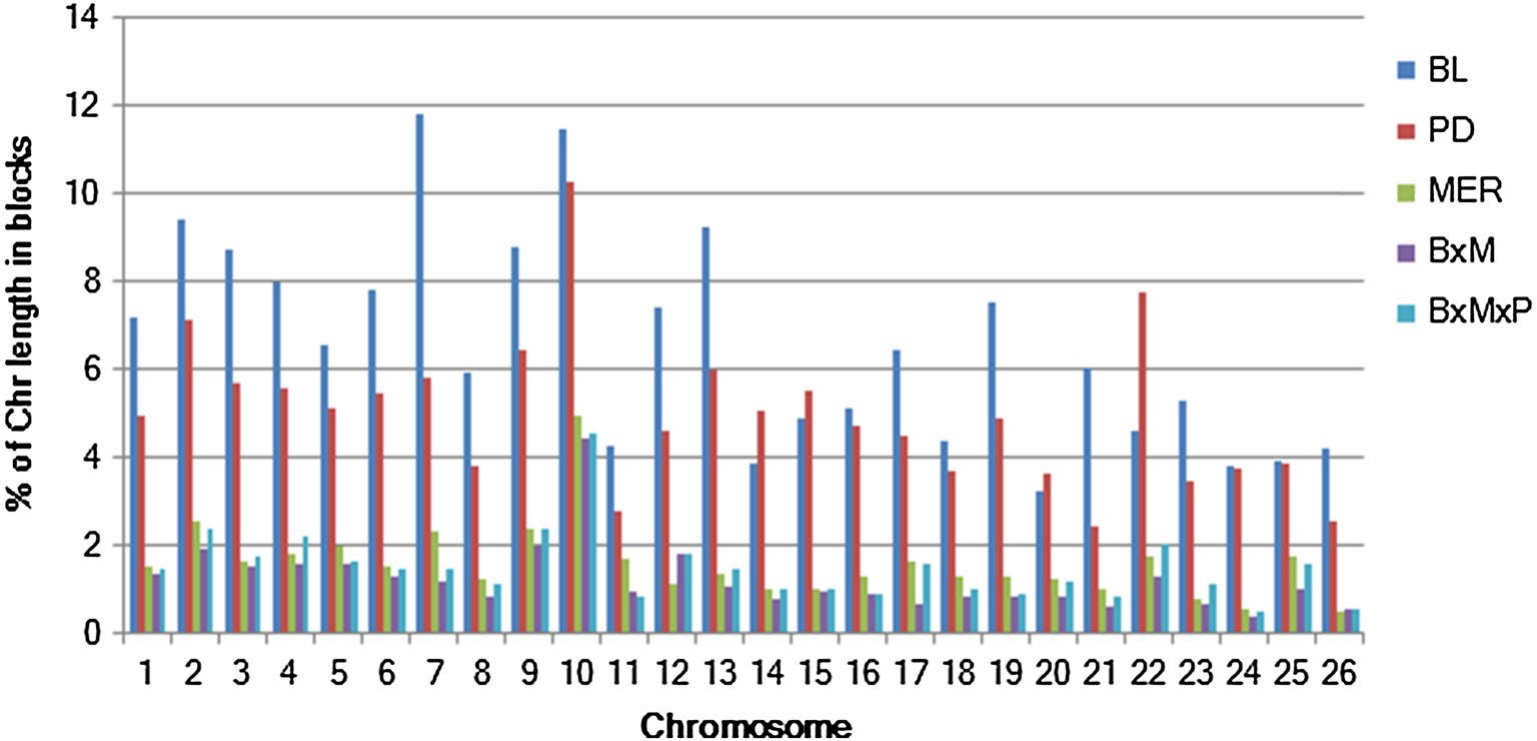
Proportion of chromosome length in haplotype blocks for each population studied

Haplotype blocks were identified using Haploview v4.2 [29] following the methods described in [30]. Table 2 summarises the distribution of haplotype blocks in the five populations. The total number of haplotype blocks ranged from 1581 (for MxB) to 2534 (for PD). Although the PD genome had the largest number of haplotype blocks, the percentage of the genome covered by haplotypes was greater for BL than for PD (177.74 Mb and 7.25 % for BL and 130.53 Mb and 5.32 % for PD). The longest block was detected on OAR10 with a length of nearly 500 kb for all populations except for BL for which the longest haplotype block was on OAR21 (~500 kb). The shortest haplotype blocks were on OAR12 for BL, MxB and MxBxP (1.95 kb), OAR5 for MER (0.06 kb) and OAR26 for PD (1.04), but these mostly just reflected the few SNP pairs that were at close distances on the array. The number of SNPs within a block varied between 2 and 12 whereas the percentage of SNPs in blocks ranged from 7.10 (for MxB) to 15.13 % (for BL). Chromosome-wise percentage coverage of blocks is in Fig. 7 and for more details (see Additional file 1: Table S6). For all populations except BL, OAR10 had the highest percentage of coverage by haplotype blocks. BL also had a large number of haplotype blocks on OAR10 but this was even larger on OAR7.

**Table 2:** Summary of haplotype blocks for each population studied

Breed	Nb of blocks	Total block length (Mb)	Proportion of chromosome length in blocks (%)	Mean block length (kb)	Median block length (kb)	Maximum block length (kb) (OAR)	Minimum block length (kb) (OAR)	Total nb of SNPs in blocks	% of SNPs in blocks	Min/max nb of SNPs in a block
BL	2511	177.74	7.25	70.78	15.89	499.13 (21)	1.95 (12)	7354	15.13	2/12
PD	2534	130.53	5.32	51.51	14.96	495.61 (10)	1.04 (26)	6769	13.93	2/10
MER	2048	42.31	1.73	20.06	12.7	493.36 (10)	0.06 (5)	4090	8.4	2/8
BxM	1581	33.73	1.37	21.34	12.65	493.36 (10)	1.95 (12)	3451	7.10	2/8
BxMxP	1810	40.17	1.64	22.19	12.72	493.36 (10)	1.95 (12)	4011	8.25	2/9

*Nb* number

### Effective population size and inbreeding coefficients

The size of *N*_*e*_ differed between populations. BL had the smallest estimated *N*_*e*_ whereas MER was much more diverse (Table 3). As expected, the two crossbred populations were also quite diverse.

**Table 3:** Average inbreeding coefficient and current effective population size for each population studied

Breed	*N*_*e*_	Inbreeding coefficient			Critical percentage		
		*F*_*1*_	*F*_*2*_	*F*_*3*_	*F*_*1*_	*F*_*2*_	*F*_*3*_
BL	140	−0.006	0.002	0.002	16.996	58.498	1.186
PD	152	−0.013	−0.001	−0.001	15.909	28.409	4.167
MER	348	−0.001	0.0004	0.0004	1.509	0	0
MxB	272	−0.085	−0.084	−0.084	0	0	0
MxBxP	152	−0.057	−0.057	−0.057	2.597	0	0

Inbreeding coefficient was calculated for each individual and then averaged across the populations

*F*_*1*_ is calculated based on the variance of the additive genotype

*F*_*2*_ is calculated based on the excess of homozygotes

*F*_*3*_ is calculated based on the correlation between uniting gametes

Critical percentage is the percentage of individuals having an *F* value >0.065

The average inbreeding estimates (*F* values) were calculated based on the SNP genotypes for all five populations (Table 3). No significant inbreeding was observed in the pure breeds and some outbreeding was observed in the crossbred populations. Estimated *F*_*2*_ values were based on the excess of homozygotes for BL, MER and PD ranged from −1.29 to 0.22, from −0.19 to 0.13, and from −1.25 to 0.23 with median values 0.09, −0.013 and 0.02, respectively, for details (see Additional file 1: Table S7).

## Discussion

In this study, we identified clear but modest genetic differences between three pure sheep breeds MER, BL and PD. Analysis of genetic diversity within these three populations (Table 1) showed that MER had the highest level of diversity with an estimated gene diversity (*H*_e_) of 0.38 and 99.86 % polymorphic SNPs. This fits well with the known history of the breed. The foundation of the Australian Merino population involves contributions from different European, Asian and African breeds [22] and, therefore, Australian Merino are a combination of strains of sheep rather than an ancient single homogenous breed. Furthermore, Australian Merinos were reported as the most diverse sheep population [22]. The higher rate of LD decay in MER confirms its high level of genetic diversity. In contrast to MER, BL and PD had lower levels of genetic diversity and a lower rate of LD decay. BL was developed in England during the 17th century from a founding stock of Dishley Leicester rams that were imported into Australia in 1871 [34]. PD sheep were developed in Australia from the Dorset sheep breed between 1937 and 1954 with the aim of breeding Dorset sheep without horns. In both cases, the populations underwent bottlenecks during breed formation which accounts for their smaller *N*_*e*_ sizes [34].

The extent and pattern of genome-wide LD are important for QTL mapping of production traits in association studies, i.e., it is the strength of LD between markers and QTL that provide the statistical power to detect associations in GWAS. In our analysis, BL and PD had a significantly higher average LD compared to MER with the latter also showing a higher rate of LD decay. Figure 6, Additional file 2: Figure S2 and Additional file 1: Table S8 clearly illustrate the differences of the two meat breeds (PD and BL), which had a higher average LD with the wool breed (MER), which had the lowest average LD. As expected the crosses (MxB and MxBxP) had an intermediate average LD compared to the meat and wool breeds. Our results indicate that LD decay was breed-specific, which is in agreement with other reports in sheep [22] and other species [35, 36]. The high LD in BL and PD is most likely attributable to the smaller *N*_*e*_ size of BL (*N*_*e*_ = 140) and PD (*N*_*e*_ = 152) in comparison to MER (*N*_*e*_ = 348). The *N*_*e*_ sizes of BL and PD are similar to those reported for Spanish Churra sheep (*N*_*e*_ = 128) [23] and for Finnsheep (*N*_*e*_ = 119 and 122) [37]. However, the *N*_*e*_ reported in our study are significantly smaller than those reported by the SheepHapMap project [1] (MER = 853, PD = 318, BL = 242) which were on average 2.1 times larger than our figures; but most of the sheep breeds analysed in the SheepHapMap project had large *N*_*e*_ [1], i.e., 600 for Spanish Churra and 795 for Finnsheep breeds. This may be due to the fact that the SheepHapMap project used fewer and unrelated animals, whereas in our study some half-sib animals were used and the average number of animals/sire ranged from 1.61 (for PD) to 2.38 (for MER). However, the rankings of *N*_*e*_ agree, with the largest and smallest *N*_*e*_ for MER and BL, respectively.

Comparison between *r*^*2*^ and *D*′ values revealed that at the same genomic distance, the average *D*′ value was larger than the average *r*^*2*^ value, which is in agreement with previous reports [22, 23]. This might be attributed to the presence of rare alleles and unobserved haplotypes that could inflate *D*′ but not *r*^*2*^ [38]. Typically, *r*^*2*^ is the preferred measure of LD in the context of QTL mapping, whereas *D*′ is the measure of choice to assess recombination patterns. Taken together, both *D*’ and *r*^2^ showed that beyond 20 Mb the average LD values for all populations are very similar to the average value of non-syntenic SNPs (see Additional file 1: Table S8), i.e., there was no LD. Previous reports on sheep with microsatellite markers reported that LD is very high and extends over a considerable range up to 20 cM and that high LD exists between marker pairs that are separated by <5 cM [22, 39]. However, a recent study by Kijas et al. [21] on sheep using SNP arrays reported that LD decays faster and at much shorter genomic distances. This is in line with what we observed here. We found that beyond 0.5 Mb, the average *r*^*2*^ and *D*′ values dropped to <0.01 and 0.45, respectively, for most breeds; the useful LD [40] did not extend beyond 0.5 Mb. Very similar results were found in a bovine whole-genome LD analysis [15]. In sheep, a recent assessment of LD in Spanish Chura also reported comparable levels of LD with an average *r*^*2*^ value of 0.329 for SNPs that are up to 10 kb apart and an average *r*^*2*^ value of 0.061 for SNPs that are separated by 200–500 kb [23]. Kijas et al. [21] reported LD over short distances using the 700 k sheep SNP array and showed that *r*^*2*^ values at 10 kb were equal to 0.186, 0.279 and 0.339, respectively, for MER, PD and BL, while we obtained *r*^*2*^ values of 027, 0.334 and 0.335. Both studies were in close agreement for BL but we obtained higher estimates of LD for MER and PD, which is due to the 50 k array including too few SNPs that are 10 kb apart to reliably estimate LD at short distances.

Comparison of our results with those of other studies on LD in other species shows that LD in sheep persists for relatively shorter genomic distances than in cattle, pigs or dogs. While LD estimates are not immediately comparable because the number of samples, the number of markers and the types of measurements vary between reports, the emerging picture is that sheep have lower LD than other domestic animals. The extent of LD in various cattle breeds is greater than that found in this study [16, 17, 41, 42]. LD analysis in six commercial pig lines showed that the average *r*^*2*^ values only dropped to <0.01 when the interval between SNPs was greater than 3 cM [43]; another study on four pig breeds, reported a rather high *r*^*2*^, i.e., between 0.19 and 0.26 for SNPs separated by 0.5 Mb [44]. Similarly, LD extends up to several Mb for dog breeds [45]. In contrast, LD in human populations extends to only tens of kb [46]. This agrees with Kijas et al. [1] who indicated that sheep have been less intensively selected than other domestic species and sampled from a larger initial gene pool. In Australia, in comparison to other livestock industries, selection pressure on sheep populations has not been as strong as for e.g. dairy cattle, which accounts for the lower levels of LD and larger *N*_*e*_ observed in this study.

**Fig. 8:**
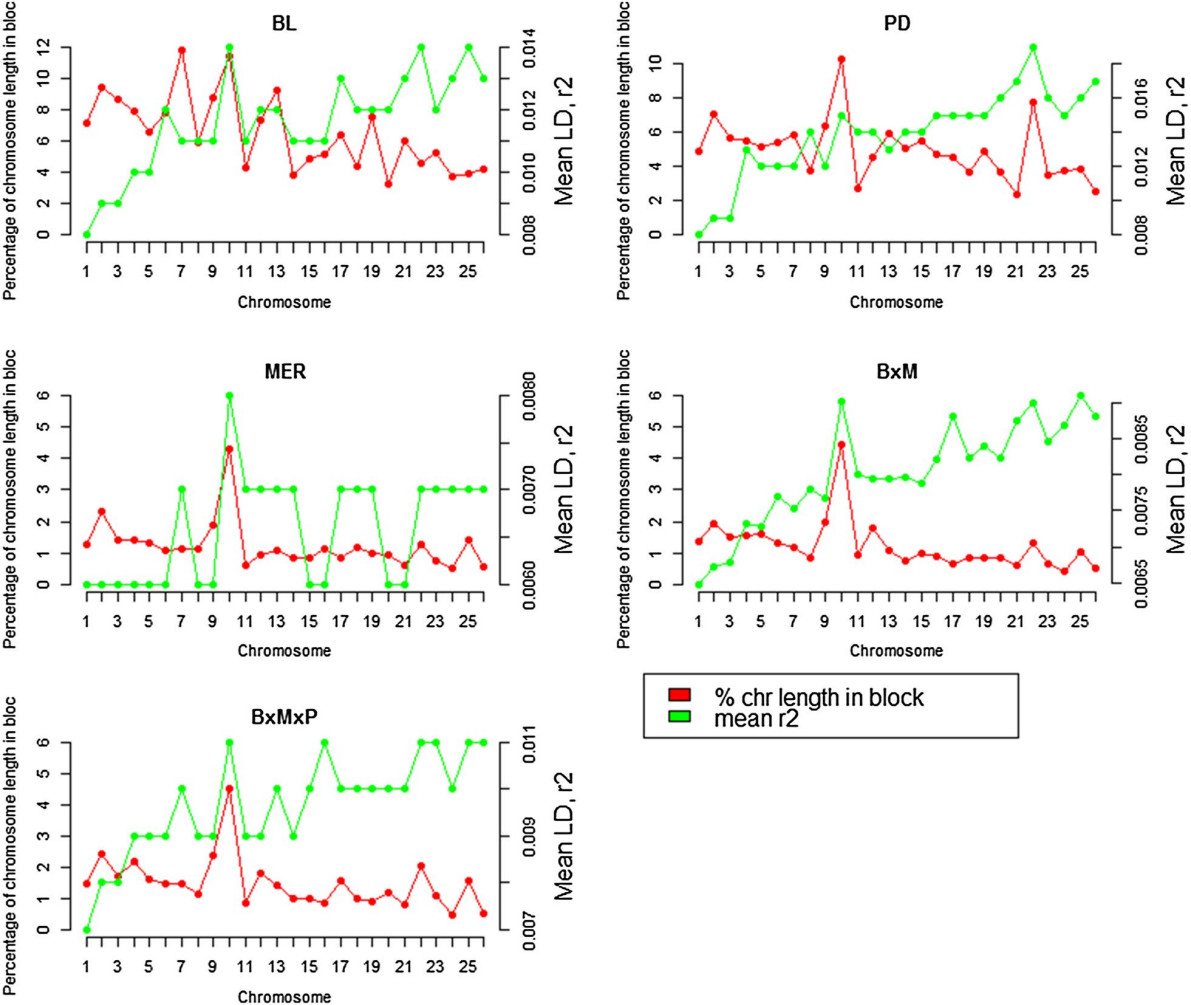
Proportion of chromosome length in haplotype blocks and average *r* ^*2*^ values for each chromosome

Chromosome-wise mean LD varied between breeds and between chromosomes (see Additional file 1: Tables S4, S5), which could be due to differences in recombination rates, heterozygosity, genetic drift and effects of selection between chromosomes and breeds [47]. Chromosome-wise average LD values were larger for BL and PD than for MER and the crossbred populations. Chromosome-wise average LD values were in agreement with the haplotype block structure and the ROH distribution across the sheep genome (Figs. 4, 8). Chromosomes that showed higher average *r*^*2*^ values also had a larger proportion of haplotype blocks and ROH. This was especially the case for OAR10, 22 and 23.

Higher levels of homozygosity and LD are expected in genomic regions that have undergone intensive selection for a particular trait (a signature of selection, as in, e.g., [1, 48]). It is also expected that these regions will have longer haplotype blocks. In the populations studied here, OAR10 was the chromosome with the largest proportion in haplotype blocks. SNP OAR10_26604546.1, which had the second largest number of occurrences in ROH counts, is on this chromosome. A total of 142 (11.15 %) animals had at least one ROH near this SNP in the region between 26.7 and 29.3 Mb. This region contains the *RXFP2* (*relaxin/insulin*-*like family peptide receptor 2*) gene, which is associated with the absence of horns in sheep [49] and for which there is also strong evidence of selection in cattle [50]. A QTL associated with horns (HO) in Soay sheep peaked at 27.1 cM on OAR10 [51, 52]. We found that BL and PD had very long haplotype blocks in this region, but MER (and the crossbred populations) had shorter blocks. PD had three long haplotype blocks of 495.6, 387 and 195 kb in the region between 28.5 and 31.1 Mb on OAR10 that contained nine, ten and six SNPs, respectively. In the same region between 28.4 and 30.4 Mb on OAR10, BL had four haplotype blocks that were 123.5, 182.8, 479 and 209.7 kb long and each contained six SNPs. In contrast, MER had four small blocks (18.2, 114.8, 8.7 and 10.5 kb) with two, four, two and two SNPs, respectively. For completeness, MxB had one block of 90 kb that contained four SNPs and MxBxP had two blocks (18.2 and 151.29 kb) that contained two and five SNPs, respectively. The significant difference in haplotype block length suggests that selection on this region has been intensive in the PD and BL breeds.

OAR2 had the largest number of ROH and a large proportion of genome within haplotype blocks. This chromosome contains SNP OAR2_119604666.1 which had the largest number of occurrences in ROH (Fig. 4). Near this SNP, 174 (13.67 %) animals had at least one ROH that spanned the region between 109.1 and 111.3 Mb. The *HERC2*-like gene i.e. *LOC101102534* (between 112.4 and 112.7 Mb) is located near this region and has been reported to affect hair colour and skin pigmentation in humans [53]. Our sheep populations were white wool sheep breeds and this region on OAR2 could be under selection.

OAR15 also has a region between 50.21 and 53.04 Mb that contains SNPs that had large numbers of occurrences in the ROH counts. A total of 109 (8.56 %) animals had at least one ROH that spanned this region. Among these 109 animals, 103 contained SNP s07957.1 (at 50 218 062) in their ROH segments. In this region, PD had a long haplotype block (399.99 kb) of 5 SNPs and BL has two long haplotype blocks of 280.63 and 413.29 kb of six SNPs each. In contrast, MER had two small haplotype blocks of 16.35 and 9.5 kb of two SNPs each. We explored a 1 Mb region (49.2 to 51.2 Mb) in each direction from the SNP s07957.1 on OAR15 and found that this region contains 39 protein coding genes, which represents about 3.6 % of the total number of genes present on OAR15 (1098 genes). The list of the genes with their description and summary functions of their human homologs is in Additional file 1: Table S9.

Genetic diversity and therefore genome-wide LD information is important for genomic selection studies. Genomic selection (GS) uses genetic markers that cover the whole genome to predict genomic estimated breeding values (GEBV). In genomic selection, the accuracy of GEBV depends largely on the heritability of the trait, its genetic architecture and the effective size of the targeted populations. The low levels of LD and high levels of diversity observed in these populations suggest that accuracies of prediction in sheep could be lower than in other populations that have higher LD under similar trait/population scenarios. As proposed by Kijas et al. [21], denser marker panels may help offset the effects of low LD. Larger datasets with more animals than the numbers used in, e.g., cattle or pig may also be required to obtain similar levels of prediction accuracy as those observed in these other species. This may be particularly true for the Merino population.

## Conclusions

We estimated and compared linkage disequilibrium (LD) and several other genetic diversity parameters, including gene diversity (*H*_*e*_) and fixation index (*F*_ST_), in five Australian sheep populations i.e. the three most economically important pure breeds and two crosses of these pure breeds. LD decayed rapidly in all populations but the rate of decay varied significantly between them. While genetic distances between breeds were relatively modest in comparison to other livestock species, the genetic diversity within Merino was high. The results of this study improve our understanding of the genetic diversity in the three main Australian sheep breeds and will be useful to perform effective GWAS studies. Our results also provide insights into the influence of selection within these breeds and provide useful knowledge that will contribute to design appropriate and successful genomic selection programs.

## Additional files

10.1186/s12711-015-0169-6 Summary statistics for the SNPs, average minor allele frequency and heterozygosity. **Table S2.** Average linkage disequilibrium (r2) between adjacent markers on the autosomes (OAR). **Table S3.** Average linkage disequilibrium (D’) between adjacent markers on the autosomes (OAR). **Table S4.** Chromosome-wise average linkage disequilibrium (D’) for each population studied. **Table S5.** Chromosome-wise average linkage disequilibrium (r2) for each population studied. **Table S6.** Summary of the chromosome-wise haplotype analysis. **Table S7.** Range of inbreeding coefficients for each population studied. **Table S8.** Mean linkage disequilibrium in the five populations at varying map distances. **Table S9.** List of the genes located in the region between 49.2 and 51.2 Mb on OAR15.
10.1186/s12711-015-0169-6 Distribution of minor allele frequency (MAF) for each population studied. The percentage of SNP is plotted for each frequency bin. **Figure S2.** Average D' values for each population.
