# Supplementary material for: Genome-wide linkage disequilibrium and genetic diversity in five populations of Australian domestic sheep
Source: Genet Sel Evol. 2015 Nov 24;47:90. doi: 10.1186/s12711-015-0169-6 (PMC4659207; doi:10.1186/s12711-015-0169-6)
Supplement: Supplementary file 1 — 10.1186/s12711-015-0169-6 Summary statistics for the SNPs, average minor allele frequency and heterozygosity. Table S2. Average linkage disequilibrium (r2) between adjacent markers on the autosomes (OAR). Table S3. Average linkage disequilibrium (D’) between adjacent markers on the autosomes (OAR). Table S4. Chromosome-wise average linkage disequilibrium (D’) for each population studied. Table S5. Chromosome-wise average linkage disequilibrium (r2) for each population studied. Table S6. Summary of the chromosome-wise haplotype analysis. Table S7. Range of inbreeding coefficients for each population studied. Table S8. Mean linkage disequilibrium in the five populations at varying map distances. Table S9. List of the genes located in the region between 49.2 and 51.2 Mb on OAR15. [file 12711_2015_169_MOESM1_ESM.docx]

**Table S1 Summary statistics for the SNPs, average minor allele frequency and heterozygosity.**

| OAR | Size (Mbp) | SNP | Mean distance between SNP(kb) | BL | | PD | | MER | | BxM | | BxMxP | |
| --- | --- | --- | --- | --- | --- | --- | --- | --- | --- | --- | --- | --- | --- |
|  |  |  |  | MAF | Het | MAF | Het | MAF | Het | MAF | Het | MAF | Het |
|  | 275.61 | 5484 | 50.26 | 0.230 | 0.308 | 0.262 | 0.345 | 0.295 | 0.383 | 0.285 | 0.405 | 0.296 | 0.404 |
|  | 248.99 | 5123 | 48.58 | 0.220 | 0.293 | 0.259 | 0.342 | 0.293 | 0.381 | 0.280 | 0.403 | 0.292 | 0.403 |
|  | 224.28 | 4637 | 48.35 | 0.224 | 0.302 | 0.255 | 0.337 | 0.289 | 0.376 | 0.282 | 0.405 | 0.293 | 0.405 |
|  | 119.26 | 2510 | 47.53 | 0.231 | 0.307 | 0.253 | 0.338 | 0.290 | 0.379 | 0.284 | 0.404 | 0.293 | 0.406 |
|  | 107.9 | 2192 | 48.8 | 0.225 | 0.302 | 0.263 | 0.345 | 0.296 | 0.384 | 0.283 | 0.404 | 0.297 | 0.402 |
|  | 117.03 | 2410 | 48.54 | 0.221 | 0.296 | 0.257 | 0.338 | 0.290 | 0.378 | 0.276 | 0.399 | 0.290 | 0.400 |
|  | 100.08 | 2094 | 47.8 | 0.217 | 0.293 | 0.262 | 0.343 | 0.297 | 0.384 | 0.286 | 0.413 | 0.297 | 0.409 |
|  | 90.7 | 1915 | 47.26 | 0.235 | 0.314 | 0.255 | 0.337 | 0.296 | 0.385 | 0.286 | 0.410 | 0.296 | 0.408 |
|  | 94.73 | 1980 | 47.84 | 0.229 | 0.305 | 0.271 | 0.356 | 0.293 | 0.380 | 0.284 | 0.404 | 0.296 | 0.403 |
|  | 86.45 | 1717 | 50.36 | 0.214 | 0.292 | 0.261 | 0.346 | 0.292 | 0.381 | 0.278 | 0.406 | 0.293 | 0.401 |
|  | 62.25 | 1103 | 56.29 | 0.230 | 0.305 | 0.255 | 0.337 | 0.273 | 0.358 | 0.274 | 0.390 | 0.286 | 0.388 |
|  | 79.1 | 1583 | 49.89 | 0.227 | 0.306 | 0.270 | 0.355 | 0.291 | 0.381 | 0.283 | 0.404 | 0.295 | 0.404 |
|  | 83.08 | 1561 | 53.24 | 0.227 | 0.297 | 0.259 | 0.341 | 0.297 | 0.381 | 0.284 | 0.404 | 0.294 | 0.408 |
|  | 62.72 | 1088 | 57.67 | 0.231 | 0.304 | 0.246 | 0.325 | 0.289 | 0.372 | 0.279 | 0.394 | 0.287 | 0.401 |
|  | 80.92 | 1553 | 52.06 | 0.232 | 0.309 | 0.252 | 0.332 | 0.289 | 0.380 | 0.284 | 0.399 | 0.288 | 0.400 |
|  | 71.72 | 1448 | 49.45 | 0.247 | 0.327 | 0.256 | 0.337 | 0.285 | 0.375 | 0.288 | 0.401 | 0.294 | 0.406 |
|  | 72.29 | 1321 | 54.64 | 0.219 | 0.289 | 0.257 | 0.340 | 0.296 | 0.385 | 0.282 | 0.407 | 0.297 | 0.410 |
|  | 68.6 | 1316 | 52.09 | 0.226 | 0.303 | 0.269 | 0.352 | 0.292 | 0.377 | 0.281 | 0.396 | 0.295 | 0.402 |
|  | 60.46 | 1152 | 52.46 | 0.236 | 0.309 | 0.247 | 0.324 | 0.289 | 0.376 | 0.283 | 0.401 | 0.286 | 0.399 |
|  | 51.18 | 1050 | 48.56 | 0.245 | 0.319 | 0.261 | 0.340 | 0.294 | 0.382 | 0.284 | 0.394 | 0.289 | 0.395 |
|  | 50.07 | 821 | 61.01 | 0.239 | 0.312 | 0.268 | 0.343 | 0.291 | 0.378 | 0.282 | 0.394 | 0.295 | 0.401 |
|  | 50.83 | 1008 | 50.47 | 0.225 | 0.303 | 0.255 | 0.337 | 0.287 | 0.377 | 0.285 | 0.406 | 0.294 | 0.407 |
|  | 62.33 | 1054 | 59.16 | 0.232 | 0.309 | 0.256 | 0.344 | 0.297 | 0.386 | 0.285 | 0.403 | 0.298 | 0.410 |
|  | 42.03 | 683 | 61.62 | 0.226 | 0.306 | 0.257 | 0.332 | 0.289 | 0.374 | 0.283 | 0.404 | 0.291 | 0.396 |
|  | 45.37 | 931 | 48.75 | 0.242 | 0.318 | 0.265 | 0.337 | 0.293 | 0.376 | 0.288 | 0.401 | 0.292 | 0.397 |
|  | 44.08 | 865 | 50.93 | 0.236 | 0.315 | 0.256 | 0.338 | 0.293 | 0.382 | 0.286 | 0.406 | 0.300 | 0.412 |

A summary of statistics for the SNP that passed quality control. within each chromosome and chromosome-wise average minor allele frequency (MAF) and heterozygosity (Het) for each breed.

**Table S2 Average linkage disequilibrium (r2) between adjacent markers on the autosomes (OAR).**

| OAR | BL | | PD | | MER | | BxM | | BxMxP | |
| --- | --- | --- | --- | --- | --- | --- | --- | --- | --- | --- |
|  | Mean | Median | Mean | Median | Mean | Median | Mean | Median | Mean | Median |
| 1 | 0.21 | 0.081 | 0.196 | 0.089 | 0.13 | 0.048 | 0.142 | 0.056 | 0.147 | 0.062 |
| 2 | 0.226 | 0.083 | 0.208 | 0.097 | 0.143 | 0.057 | 0.156 | 0.060 | 0.153 | 0.065 |
| 3 | 0.222 | 0.084 | 0.204 | 0.088 | 0.134 | 0.055 | 0.150 | 0.059 | 0.148 | 0.062 |
| 4 | 0.211 | 0.08 | 0.202 | 0.094 | 0.137 | 0.056 | 0.146 | 0.055 | 0.151 | 0.061 |
| 5 | 0.212 | 0.075 | 0.192 | 0.082 | 0.124 | 0.048 | 0.134 | 0.046 | 0.143 | 0.056 |
| 6 | 0.21 | 0.079 | 0.19 | 0.087 | 0.124 | 0.05 | 0.142 | 0.057 | 0.141 | 0.06 |
| 7 | 0.229 | 0.078 | 0.205 | 0.089 | 0.129 | 0.053 | 0.153 | 0.064 | 0.155 | 0.067 |
| 8 | 0.216 | 0.084 | 0.196 | 0.085 | 0.128 | 0.051 | 0.142 | 0.061 | 0.144 | 0.061 |
| 9 | 0.211 | 0.077 | 0.187 | 0.081 | 0.128 | 0.049 | 0.137 | 0.056 | 0.141 | 0.061 |
| 10 | 0.246 | 0.087 | 0.21 | 0.088 | 0.148 | 0.054 | 0.166 | 0.061 | 0.162 | 0.07 |
| 11 | 0.175 | 0.058 | 0.173 | 0.067 | 0.111 | 0.038 | 0.114 | 0.037 | 0.123 | 0.043 |
| 12 | 0.219 | 0.083 | 0.182 | 0.08 | 0.126 | 0.044 | 0.138 | 0.055 | 0.145 | 0.06 |
| 13 | 0.209 | 0.069 | 0.198 | 0.084 | 0.137 | 0.052 | 0.139 | 0.049 | 0.142 | 0.055 |
| 14 | 0.184 | 0.054 | 0.182 | 0.069 | 0.11 | 0.041 | 0.117 | 0.041 | 0.116 | 0.037 |
| 15 | 0.195 | 0.072 | 0.2 | 0.087 | 0.124 | 0.049 | 0.130 | 0.049 | 0.138 | 0.056 |
| 16 | 0.185 | 0.073 | 0.192 | 0.088 | 0.121 | 0.047 | 0.124 | 0.051 | 0.138 | 0.063 |
| 17 | 0.218 | 0.078 | 0.188 | 0.082 | 0.117 | 0.047 | 0.142 | 0.051 | 0.135 | 0.049 |
| 18 | 0.195 | 0.07 | 0.188 | 0.09 | 0.127 | 0.049 | 0.131 | 0.046 | 0.14 | 0.054 |
| 19 | 0.204 | 0.072 | 0.192 | 0.077 | 0.137 | 0.049 | 0.141 | 0.051 | 0.141 | 0.051 |
| 20 | 0.18 | 0.067 | 0.166 | 0.074 | 0.109 | 0.043 | 0.115 | 0.045 | 0.117 | 0.042 |
| 21 | 0.188 | 0.064 | 0.182 | 0.069 | 0.108 | 0.036 | 0.125 | 0.046 | 0.122 | 0.043 |
| 22 | 0.206 | 0.085 | 0.196 | 0.075 | 0.118 | 0.04 | 0.133 | 0.049 | 0.142 | 0.064 |
| 23 | 0.174 | 0.067 | 0.184 | 0.079 | 0.108 | 0.04 | 0.107 | 0.043 | 0.118 | 0.042 |
| 24 | 0.195 | 0.064 | 0.162 | 0.06 | 0.109 | 0.035 | 0.118 | 0.040 | 0.122 | 0.041 |
| 25 | 0.197 | 0.073 | 0.195 | 0.087 | 0.114 | 0.044 | 0.124 | 0.046 | 0.136 | 0.056 |
| 26 | 0.184 | 0.075 | 0.176 | 0.072 | 0.107 | 0.04 | 0.115 | 0.045 | 0.12 | 0.054 |

**Table S3 Average linkage disequilibrium (D’) between adjacent markers on the autosomes (OAR).**

| OAR | BL | | PD | | MER | | BxM | | BxMxP | |
| --- | --- | --- | --- | --- | --- | --- | --- | --- | --- | --- |
|  | Mean | Median | Mean | Median | Mean | Median | Mean | Median | Mean | Median |
|  | 0.732 | 0.883 | 0.701 | 0.836 | 0.543 | 0.517 | 0.584 | 0.592 | 0.576 | 0.581 |
|  | 0.755 | 0.926 | 0.733 | 0.896 | 0.568 | 0.564 | 0.604 | 0.627 | 0.587 | 0.599 |
|  | 0.749 | 0.912 | 0.73 | 0.889 | 0.567 | 0.564 | 0.607 | 0.638 | 0.586 | 0.598 |
|  | 0.733 | 0.88 | 0.728 | 0.874 | 0.563 | 0.546 | 0.592 | 0.592 | 0.583 | 0.594 |
|  | 0.736 | 0.897 | 0.696 | 0.816 | 0.543 | 0.507 | 0.559 | 0.541 | 0.567 | 0.556 |
|  | 0.743 | 0.9 | 0.722 | 0.86 | 0.546 | 0.524 | 0.594 | 0.623 | 0.583 | 0.593 |
|  | 0.752 | 0.913 | 0.727 | 0.882 | 0.536 | 0.494 | 0.599 | 0.615 | 0.584 | 0.603 |
|  | 0.74 | 0.885 | 0.707 | 0.85 | 0.532 | 0.502 | 0.567 | 0.562 | 0.558 | 0.548 |
|  | 0.753 | 0.905 | 0.698 | 0.846 | 0.543 | 0.53 | 0.580 | 0.571 | 0.572 | 0.565 |
|  | 0.765 | 0.921 | 0.722 | 0.889 | 0.571 | 0.563 | 0.598 | 0.613 | 0.59 | 0.604 |
|  | 0.719 | 0.859 | 0.696 | 0.823 | 0.547 | 0.519 | 0.546 | 0.558 | 0.547 | 0.548 |
|  | 0.752 | 0.905 | 0.687 | 0.809 | 0.542 | 0.53 | 0.575 | 0.583 | 0.563 | 0.568 |
|  | 0.721 | 0.886 | 0.716 | 0.862 | 0.548 | 0.521 | 0.556 | 0.525 | 0.555 | 0.552 |
|  | 0.723 | 0.88 | 0.714 | 0.849 | 0.523 | 0.493 | 0.546 | 0.514 | 0.53 | 0.491 |
|  | 0.726 | 0.879 | 0.744 | 0.902 | 0.544 | 0.516 | 0.560 | 0.544 | 0.569 | 0.566 |
|  | 0.716 | 0.865 | 0.718 | 0.866 | 0.549 | 0.525 | 0.555 | 0.539 | 0.573 | 0.569 |
|  | 0.718 | 0.858 | 0.694 | 0.805 | 0.516 | 0.464 | 0.567 | 0.561 | 0.549 | 0.517 |
|  | 0.713 | 0.859 | 0.701 | 0.841 | 0.542 | 0.52 | 0.563 | 0.544 | 0.564 | 0.562 |
|  | 0.735 | 0.901 | 0.747 | 0.899 | 0.547 | 0.525 | 0.571 | 0.568 | 0.577 | 0.588 |
|  | 0.709 | 0.855 | 0.683 | 0.803 | 0.506 | 0.464 | 0.533 | 0.499 | 0.532 | 0.513 |
|  | 0.718 | 0.852 | 0.69 | 0.834 | 0.481 | 0.419 | 0.542 | 0.513 | 0.531 | 0.512 |
|  | 0.724 | 0.873 | 0.72 | 0.85 | 0.529 | 0.478 | 0.565 | 0.554 | 0.586 | 0.595 |
|  | 0.707 | 0.827 | 0.679 | 0.78 | 0.505 | 0.457 | 0.523 | 0.493 | 0.516 | 0.473 |
|  | 0.698 | 0.821 | 0.667 | 0.762 | 0.503 | 0.47 | 0.509 | 0.457 | 0.532 | 0.506 |
|  | 0.728 | 0.882 | 0.707 | 0.834 | 0.517 | 0.464 | 0.551 | 0.510 | 0.551 | 0.552 |
|  | 0.723 | 0.872 | 0.696 | 0.82 | 0.524 | 0.479 | 0.540 | 0.499 | 0.551 | 0.55 |

**Table S4 Chromosome-wise average linkage disequilibrium (D’) for each population studied.**

| OAR | BL | | PD | | MER | | BxM | | BxMxP | |
| --- | --- | --- | --- | --- | --- | --- | --- | --- | --- | --- |
|  | Mean | Median | Mean | Median | Mean | Median | Mean | Median | Mean | Median |
|  | 0.276 | 0.152 | 0.222 | 0.127 | 0.15 | 0.094 | 0.176 | 0.110 | 0.176 | 0.114 |
|  | 0.299 | 0.168 | 0.236 | 0.133 | 0.151 | 0.094 | 0.181 | 0.113 | 0.184 | 0.120 |
|  | 0.288 | 0.16 | 0.241 | 0.135 | 0.156 | 0.097 | 0.183 | 0.114 | 0.188 | 0.122 |
|  | 0.278 | 0.159 | 0.262 | 0.155 | 0.161 | 0.1 | 0.182 | 0.114 | 0.189 | 0.124 |
|  | 0.299 | 0.168 | 0.238 | 0.144 | 0.158 | 0.098 | 0.183 | 0.115 | 0.192 | 0.125 |
|  | 0.312 | 0.182 | 0.251 | 0.146 | 0.158 | 0.1 | 0.195 | 0.121 | 0.200 | 0.131 |
|  | 0.322 | 0.187 | 0.252 | 0.145 | 0.152 | 0.096 | 0.184 | 0.115 | 0.195 | 0.128 |
|  | 0.283 | 0.165 | 0.266 | 0.159 | 0.153 | 0.098 | 0.176 | 0.115 | 0.186 | 0.125 |
|  | 0.299 | 0.174 | 0.236 | 0.137 | 0.159 | 0.099 | 0.184 | 0.117 | 0.184 | 0.123 |
|  | 0.32 | 0.186 | 0.267 | 0.157 | 0.165 | 0.103 | 0.197 | 0.123 | 0.203 | 0.135 |
|  | 0.304 | 0.172 | 0.282 | 0.164 | 0.199 | 0.116 | 0.208 | 0.127 | 0.214 | 0.134 |
|  | 0.29 | 0.169 | 0.253 | 0.151 | 0.161 | 0.102 | 0.186 | 0.118 | 0.190 | 0.127 |
|  | 0.3 | 0.175 | 0.264 | 0.152 | 0.167 | 0.102 | 0.186 | 0.117 | 0.196 | 0.132 |
|  | 0.317 | 0.179 | 0.282 | 0.167 | 0.168 | 0.104 | 0.196 | 0.122 | 0.208 | 0.133 |
|  | 0.298 | 0.169 | 0.28 | 0.166 | 0.168 | 0.105 | 0.189 | 0.119 | 0.201 | 0.132 |
|  | 0.281 | 0.16 | 0.287 | 0.175 | 0.176 | 0.11 | 0.183 | 0.117 | 0.203 | 0.137 |
|  | 0.308 | 0.184 | 0.263 | 0.163 | 0.158 | 0.102 | 0.195 | 0.123 | 0.193 | 0.130 |
|  | 0.296 | 0.176 | 0.264 | 0.16 | 0.173 | 0.107 | 0.198 | 0.124 | 0.200 | 0.131 |
|  | 0.31 | 0.175 | 0.306 | 0.179 | 0.175 | 0.11 | 0.196 | 0.123 | 0.211 | 0.139 |
|  | 0.299 | 0.172 | 0.275 | 0.173 | 0.161 | 0.102 | 0.185 | 0.118 | 0.203 | 0.135 |
|  | 0.305 | 0.178 | 0.286 | 0.175 | 0.166 | 0.106 | 0.192 | 0.124 | 0.200 | 0.132 |
|  | 0.299 | 0.177 | 0.29 | 0.184 | 0.168 | 0.107 | 0.194 | 0.124 | 0.208 | 0.139 |
|  | 0.294 | 0.175 | 0.266 | 0.162 | 0.166 | 0.104 | 0.187 | 0.121 | 0.200 | 0.134 |
|  | 0.307 | 0.183 | 0.284 | 0.167 | 0.181 | 0.111 | 0.200 | 0.125 | 0.210 | 0.138 |
|  | 0.31 | 0.178 | 0.281 | 0.178 | 0.176 | 0.109 | 0.197 | 0.123 | 0.213 | 0.140 |
|  | 0.293 | 0.172 | 0.276 | 0.173 | 0.168 | 0.105 | 0.185 | 0.121 | 0.198 | 0.134 |

**Table S5 Chromosome-wise average linkage disequilibrium (r2) for each population studied.**

| OAR | BL | | PD | | MER | | BxM | | BxMxP | |
| --- | --- | --- | --- | --- | --- | --- | --- | --- | --- | --- |
|  | Mean | Median | Mean | Median | Mean | Median | Mean | Median | Mean | Median |
|  | 0.008 | 0.003 | 0.008 | 0.003 | 0.005 | 0.002 | 0.006 | 0.003 | 0.008 | 0.003 |
|  | 0.009 | 0.003 | 0.009 | 0.003 | 0.006 | 0.002 | 0.007 | 0.003 | 0.008 | 0.004 |
|  | 0.009 | 0.003 | 0.009 | 0.003 | 0.006 | 0.002 | 0.007 | 0.003 | 0.009 | 0.004 |
|  | 0.01 | 0.003 | 0.013 | 0.004 | 0.006 | 0.003 | 0.007 | 0.003 | 0.010 | 0.004 |
|  | 0.01 | 0.003 | 0.012 | 0.004 | 0.006 | 0.003 | 0.007 | 0.003 | 0.010 | 0.004 |
|  | 0.012 | 0.004 | 0.012 | 0.004 | 0.006 | 0.003 | 0.008 | 0.003 | 0.010 | 0.004 |
|  | 0.011 | 0.003 | 0.012 | 0.004 | 0.006 | 0.003 | 0.008 | 0.003 | 0.011 | 0.004 |
|  | 0.011 | 0.004 | 0.014 | 0.004 | 0.007 | 0.003 | 0.008 | 0.003 | 0.010 | 0.004 |
|  | 0.011 | 0.004 | 0.012 | 0.004 | 0.006 | 0.003 | 0.008 | 0.003 | 0.010 | 0.004 |
|  | 0.014 | 0.004 | 0.015 | 0.004 | 0.007 | 0.003 | 0.009 | 0.003 | 0.012 | 0.005 |
|  | 0.011 | 0.003 | 0.014 | 0.004 | 0.007 | 0.003 | 0.008 | 0.003 | 0.010 | 0.004 |
|  | 0.012 | 0.004 | 0.013 | 0.004 | 0.007 | 0.003 | 0.008 | 0.003 | 0.010 | 0.004 |
|  | 0.012 | 0.004 | 0.013 | 0.004 | 0.007 | 0.003 | 0.008 | 0.003 | 0.011 | 0.004 |
|  | 0.011 | 0.003 | 0.014 | 0.004 | 0.007 | 0.003 | 0.008 | 0.003 | 0.010 | 0.004 |
|  | 0.011 | 0.003 | 0.014 | 0.004 | 0.007 | 0.003 | 0.008 | 0.003 | 0.011 | 0.004 |
|  | 0.011 | 0.004 | 0.015 | 0.004 | 0.007 | 0.003 | 0.008 | 0.003 | 0.012 | 0.005 |
|  | 0.013 | 0.004 | 0.015 | 0.004 | 0.007 | 0.003 | 0.009 | 0.003 | 0.011 | 0.004 |
|  | 0.012 | 0.004 | 0.015 | 0.004 | 0.007 | 0.003 | 0.008 | 0.003 | 0.011 | 0.004 |
|  | 0.012 | 0.004 | 0.014 | 0.004 | 0.008 | 0.003 | 0.008 | 0.003 | 0.011 | 0.004 |
|  | 0.012 | 0.004 | 0.016 | 0.005 | 0.007 | 0.003 | 0.008 | 0.003 | 0.011 | 0.005 |
|  | 0.013 | 0.004 | 0.017 | 0.005 | 0.007 | 0.003 | 0.009 | 0.003 | 0.011 | 0.004 |
|  | 0.014 | 0.004 | 0.019 | 0.005 | 0.007 | 0.003 | 0.009 | 0.003 | 0.012 | 0.005 |
|  | 0.012 | 0.004 | 0.016 | 0.004 | 0.007 | 0.003 | 0.008 | 0.003 | 0.012 | 0.005 |
|  | 0.013 | 0.004 | 0.014 | 0.004 | 0.008 | 0.003 | 0.009 | 0.003 | 0.011 | 0.004 |
|  | 0.014 | 0.004 | 0.017 | 0.005 | 0.008 | 0.003 | 0.009 | 0.003 | 0.012 | 0.005 |
|  | 0.013 | 0.004 | 0.018 | 0.005 | 0.007 | 0.003 | 0.009 | 0.003 | 0.012 | 0.005 |

**Table S6 Summary of the chromosome-wise haplotype analysis.**

| Breed | Chromosome | Number of Blocks | Total block length (kb) | % of chromosome length in blocks | Min block length (kb) | Max block length (kb) | Number of SNP in blocks | % of SNP in blocks |
| --- | --- | --- | --- | --- | --- | --- | --- | --- |
| BL | 1 | 303 | 19711.34 | 7.15 | 2.97 | 462.71 | 861 | 15.70 |
| BL | 2 | 289 | 23471.07 | 9.43 | 3.66 | 481.76 | 895 | 17.47 |
| BL | 3 | 258 | 19500.66 | 8.69 | 3.27 | 493.11 | 771 | 16.63 |
| BL | 4 | 134 | 9479.03 | 7.95 | 3.91 | 373.78 | 405 | 16.14 |
| BL | 5 | 116 | 7058.86 | 6.54 | 4.76 | 489.39 | 325 | 14.83 |
| BL | 6 | 118 | 9166.13 | 7.83 | 4.97 | 475.06 | 357 | 14.81 |
| BL | 7 | 114 | 11824.49 | 11.82 | 6.05 | 498.58 | 393 | 18.77 |
| BL | 8 | 99 | 5366.45 | 5.92 | 2.62 | 408.97 | 272 | 14.20 |
| BL | 9 | 120 | 8296.02 | 8.76 | 4.18 | 470.45 | 349 | 17.63 |
| BL | 10 | 99 | 9910.07 | 11.46 | 3.95 | 493.36 | 315 | 18.35 |
| BL | 11 | 56 | 2658.05 | 4.27 | 3.87 | 283.53 | 146 | 13.24 |
| BL | 12 | 99 | 5834.46 | 7.38 | 1.95 | 394.87 | 274 | 17.31 |
| BL | 13 | 77 | 7686.51 | 9.25 | 3.75 | 476.38 | 262 | 16.78 |
| BL | 14 | 44 | 2424.21 | 3.87 | 3.56 | 449.88 | 118 | 10.85 |
| BL | 15 | 64 | 3939.06 | 4.87 | 2.13 | 458.02 | 171 | 11.01 |
| BL | 16 | 65 | 3689.49 | 5.14 | 4.62 | 304.22 | 179 | 12.36 |
| BL | 17 | 63 | 4632.88 | 6.41 | 4.81 | 428.87 | 183 | 13.85 |
| BL | 18 | 57 | 3002.75 | 4.38 | 5.08 | 243.19 | 155 | 11.78 |
| BL | 19 | 54 | 4536.79 | 7.50 | 5.73 | 436.40 | 164 | 14.24 |
| BL | 20 | 43 | 1651.83 | 3.23 | 6.33 | 202.95 | 111 | 10.57 |
| BL | 21 | 38 | 3020.14 | 6.03 | 4.88 | 499.13 | 106 | 12.91 |
| BL | 22 | 58 | 2353.13 | 4.63 | 2.32 | 300.62 | 154 | 15.28 |
| BL | 23 | 44 | 3286.40 | 5.27 | 6.60 | 370.09 | 121 | 11.48 |
| BL | 24 | 32 | 1591.10 | 3.79 | 4.38 | 413.60 | 83 | 12.15 |
| BL | 25 | 40 | 1783.64 | 3.93 | 5.51 | 408.69 | 105 | 11.28 |
| BL | 26 | 27 | 1865.04 | 4.23 | 3.05 | 386.45 | 79 | 9.13 |
| BxM | 1 | 191 | 3779.53 | 1.37 | 3.10 | 309.66 | 416 | 7.59 |
| BxM | 2 | 195 | 4768.56 | 1.92 | 4.84 | 473.56 | 432 | 8.43 |
| BxM | 3 | 148 | 3352.31 | 1.49 | 4.05 | 259.01 | 333 | 7.18 |
| BxM | 4 | 87 | 1847.98 | 1.55 | 2.89 | 226.81 | 191 | 7.61 |
| BxM | 5 | 72 | 1717.52 | 1.59 | 3.73 | 286.25 | 159 | 7.25 |
| BxM | 6 | 82 | 1536.67 | 1.31 | 3.72 | 207.77 | 178 | 7.39 |
| BxM | 7 | 66 | 1188.49 | 1.19 | 2.20 | 172.38 | 141 | 6.73 |
| BxM | 8 | 61 | 766.88 | 0.85 | 4.16 | 20.91 | 126 | 6.58 |
| BxM | 9 | 72 | 1891.31 | 2.00 | 3.66 | 286.93 | 158 | 7.98 |
| BxM | 10 | 68 | 3831.75 | 4.43 | 3.95 | 493.36 | 171 | 9.96 |
| BxM | 11 | 30 | 592.28 | 0.95 | 5.63 | 152.43 | 64 | 5.80 |
| BxM | 12 | 58 | 1433.21 | 1.81 | 1.95 | 378.21 | 127 | 8.02 |
| BxM | 13 | 47 | 888.59 | 1.07 | 3.75 | 144.14 | 103 | 6.60 |
| BxM | 14 | 32 | 472.07 | 0.75 | 5.21 | 36.06 | 67 | 6.16 |
| BxM | 15 | 38 | 782.09 | 0.97 | 2.13 | 357.20 | 79 | 5.09 |
| BxM | 16 | 34 | 651.00 | 0.91 | 5.62 | 178.25 | 77 | 5.32 |
| BxM | 17 | 33 | 482.42 | 0.67 | 4.36 | 122.78 | 68 | 5.15 |
| BxM | 18 | 42 | 571.39 | 0.83 | 5.08 | 99.97 | 87 | 6.61 |
| BxM | 19 | 31 | 504.67 | 0.83 | 5.73 | 110.19 | 66 | 5.73 |
| BxM | 20 | 30 | 440.24 | 0.86 | 6.33 | 99.75 | 64 | 6.10 |
| BxM | 21 | 25 | 306.19 | 0.61 | 2.84 | 24.91 | 53 | 6.46 |
| BxM | 22 | 43 | 670.58 | 1.32 | 2.32 | 82.99 | 91 | 9.03 |
| BxM | 23 | 28 | 397.01 | 0.64 | 6.60 | 26.49 | 59 | 5.60 |
| BxM | 24 | 16 | 166.84 | 0.40 | 4.38 | 18.32 | 33 | 4.83 |
| BxM | 25 | 32 | 460.39 | 1.01 | 5.35 | 85.22 | 68 | 7.30 |
| BxM | 26 | 20 | 233.02 | 0.53 | 3.05 | 17.88 | 40 | 4.62 |
| BxMxP | 1 | 230 | 4065.53 | 1.48 | 3.10 | 238.07 | 502 | 9.15 |
| BxMxP | 2 | 205 | 5981.95 | 2.40 | 3.66 | 473.56 | 474 | 9.25 |
| BxMxP | 3 | 160 | 3879.36 | 1.73 | 4.10 | 464.91 | 365 | 7.87 |
| BxMxP | 4 | 98 | 2624.25 | 2.20 | 2.89 | 265.74 | 227 | 9.04 |
| BxMxP | 5 | 83 | 1733.78 | 1.61 | 3.73 | 286.25 | 180 | 8.21 |
| BxMxP | 6 | 94 | 1726.91 | 1.48 | 4.80 | 281.31 | 200 | 8.30 |
| BxMxP | 7 | 84 | 1458.43 | 1.46 | 2.20 | 172.38 | 178 | 8.50 |
| BxMxP | 8 | 80 | 1035.34 | 1.14 | 2.62 | 20.91 | 163 | 8.51 |
| BxMxP | 9 | 87 | 2251.14 | 2.38 | 4.18 | 286.93 | 197 | 9.95 |
| BxMxP | 10 | 80 | 3908.01 | 4.52 | 3.95 | 493.36 | 205 | 11.94 |
| BxMxP | 11 | 34 | 525.28 | 0.84 | 5.55 | 175.09 | 72 | 6.53 |
| BxMxP | 12 | 69 | 1440.31 | 1.82 | 1.95 | 249.39 | 150 | 9.48 |
| BxMxP | 13 | 59 | 1200.42 | 1.44 | 3.75 | 238.55 | 130 | 8.33 |
| BxMxP | 14 | 38 | 637.37 | 1.02 | 3.56 | 118.51 | 82 | 7.54 |
| BxMxP | 15 | 54 | 805.03 | 0.99 | 3.39 | 135.05 | 113 | 7.28 |
| BxMxP | 16 | 38 | 630.65 | 0.88 | 3.41 | 103.76 | 83 | 5.73 |
| BxMxP | 17 | 41 | 1134.17 | 1.57 | 4.81 | 162.27 | 97 | 7.34 |
| BxMxP | 18 | 46 | 698.30 | 1.02 | 5.08 | 186.87 | 95 | 7.22 |
| BxMxP | 19 | 35 | 535.18 | 0.89 | 6.50 | 53.96 | 73 | 6.34 |
| BxMxP | 20 | 29 | 614.82 | 1.20 | 6.36 | 185.01 | 63 | 6.00 |
| BxMxP | 21 | 27 | 409.42 | 0.82 | 2.84 | 99.92 | 58 | 7.06 |
| BxMxP | 22 | 45 | 1026.98 | 2.02 | 2.32 | 229.60 | 99 | 9.82 |
| BxMxP | 23 | 23 | 696.40 | 1.12 | 6.60 | 393.23 | 51 | 4.84 |
| BxMxP | 24 | 18 | 201.89 | 0.48 | 4.38 | 27.11 | 39 | 5.71 |
| BxMxP | 25 | 33 | 713.25 | 1.57 | 5.35 | 167.48 | 75 | 8.06 |
| BxMxP | 26 | 20 | 232.48 | 0.53 | 3.05 | 19.53 | 40 | 4.62 |
| MER | 1 | 250 | 4202.71 | 1.52 | 1.39 | 168.04 | 535 | 9.76 |
| MER | 2 | 257 | 6358.13 | 2.55 | 3.66 | 473.56 | 577 | 11.26 |
| MER | 3 | 190 | 3678.32 | 1.64 | 3.99 | 246.35 | 418 | 9.01 |
| MER | 4 | 106 | 2160.60 | 1.81 | 2.89 | 226.81 | 231 | 9.20 |
| MER | 5 | 98 | 2108.58 | 1.95 | 0.06 | 310.86 | 217 | 9.90 |
| MER | 6 | 102 | 1788.89 | 1.53 | 3.72 | 228.13 | 218 | 9.05 |
| MER | 7 | 103 | 2329.15 | 2.33 | 2.20 | 322.01 | 225 | 10.74 |
| MER | 8 | 78 | 1102.24 | 1.22 | 2.62 | 80.23 | 163 | 8.51 |
| MER | 9 | 97 | 2254.96 | 2.38 | 3.66 | 392.39 | 216 | 10.91 |
| MER | 10 | 88 | 4255.97 | 4.92 | 2.46 | 493.36 | 223 | 12.99 |
| MER | 11 | 35 | 1054.53 | 1.69 | 5.78 | 312.82 | 84 | 7.62 |
| MER | 12 | 67 | 901.16 | 1.14 | 1.95 | 86.44 | 140 | 8.84 |
| MER | 13 | 60 | 1137.76 | 1.37 | 3.75 | 188.44 | 131 | 8.39 |
| MER | 14 | 38 | 643.11 | 1.03 | 0.16 | 118.51 | 82 | 7.54 |
| MER | 15 | 58 | 813.33 | 1.01 | 2.13 | 107.41 | 120 | 7.73 |
| MER | 16 | 47 | 919.77 | 1.28 | 4.38 | 178.25 | 104 | 7.18 |
| MER | 17 | 47 | 1186.32 | 1.64 | 3.36 | 275.76 | 106 | 8.02 |
| MER | 18 | 53 | 872.17 | 1.27 | 5.08 | 129.02 | 115 | 8.74 |
| MER | 19 | 52 | 773.47 | 1.28 | 5.73 | 77.46 | 107 | 9.29 |
| MER | 20 | 39 | 642.83 | 1.26 | 3.21 | 119.13 | 84 | 8.00 |
| MER | 21 | 28 | 500.87 | 1.00 | 2.84 | 178.05 | 61 | 7.43 |
| MER | 22 | 45 | 888.33 | 1.75 | 2.32 | 229.60 | 98 | 9.72 |
| MER | 23 | 33 | 500.19 | 0.80 | 6.27 | 62.12 | 71 | 6.74 |
| MER | 24 | 21 | 234.09 | 0.56 | 4.38 | 18.98 | 42 | 6.15 |
| MER | 25 | 36 | 796.73 | 1.76 | 4.20 | 308.34 | 82 | 8.81 |
| MER | 26 | 20 | 207.54 | 0.47 | 1.04 | 17.69 | 40 | 4.62 |
| PD | 1 | 321 | 13623.07 | 4.94 | 2.97 | 446.63 | 810 | 14.77 |
| PD | 2 | 294 | 17712.33 | 7.11 | 3.16 | 495.44 | 818 | 15.97 |
| PD | 3 | 233 | 12716.85 | 5.67 | 3.27 | 485.56 | 643 | 13.87 |
| PD | 4 | 126 | 6621.48 | 5.55 | 2.89 | 395.86 | 350 | 13.94 |
| PD | 5 | 126 | 5551.59 | 5.15 | 3.73 | 411.00 | 320 | 14.60 |
| PD | 6 | 118 | 6370.30 | 5.44 | 4.80 | 463.58 | 331 | 13.73 |
| PD | 7 | 129 | 5827.82 | 5.82 | 3.49 | 279.39 | 337 | 16.09 |
| PD | 8 | 95 | 3430.33 | 3.78 | 4.16 | 303.84 | 237 | 12.38 |
| PD | 9 | 114 | 6072.24 | 6.41 | 4.18 | 396.99 | 300 | 15.15 |
| PD | 10 | 106 | 8875.84 | 10.27 | 2.46 | 495.61 | 326 | 18.99 |
| PD | 11 | 53 | 1716.96 | 2.76 | 5.13 | 220.13 | 125 | 11.33 |
| PD | 12 | 90 | 3637.74 | 4.60 | 1.95 | 362.30 | 230 | 14.53 |
| PD | 13 | 73 | 4977.02 | 5.99 | 1.67 | 414.73 | 216 | 13.84 |
| PD | 14 | 57 | 3160.23 | 5.04 | 3.56 | 494.13 | 149 | 13.69 |
| PD | 15 | 79 | 4468.57 | 5.52 | 2.13 | 439.89 | 210 | 13.52 |
| PD | 16 | 55 | 3377.67 | 4.71 | 3.41 | 448.60 | 159 | 10.98 |
| PD | 17 | 59 | 3266.64 | 4.52 | 4.81 | 335.30 | 157 | 11.88 |
| PD | 18 | 63 | 2526.58 | 3.68 | 5.08 | 341.82 | 154 | 11.70 |
| PD | 19 | 55 | 2961.80 | 4.90 | 5.73 | 341.54 | 145 | 12.59 |
| PD | 20 | 47 | 1870.49 | 3.65 | 3.21 | 332.01 | 114 | 10.86 |
| PD | 21 | 37 | 1212.40 | 2.42 | 2.84 | 212.17 | 92 | 11.21 |
| PD | 22 | 59 | 3933.26 | 7.74 | 2.32 | 441.51 | 174 | 17.26 |
| PD | 23 | 41 | 2166.66 | 3.48 | 6.91 | 486.27 | 105 | 9.96 |
| PD | 24 | 30 | 1577.27 | 3.75 | 4.38 | 476.83 | 74 | 10.83 |
| PD | 25 | 43 | 1744.98 | 3.85 | 6.02 | 205.28 | 116 | 12.46 |
| PD | 26 | 31 | 1126.87 | 2.56 | 1.04 | 196.46 | 77 | 8.90 |

**Table S7 Range of inbreeding coefficients for each population studied.**

| Breed | *F_1_* | | *F_2_* | | *F_3_* | |
| --- | --- | --- | --- | --- | --- | --- |
|  | *Min* | *Max* | *Min* | *Max* | *Min* | *Max* |
| BL | -0.22 | 1.18 | -1.3 | 0.29 | -0.07 | 0.13 |
| PD | -0.24 | 0.68 | -1.25 | 0.23 | -0.31 | 0.1 |
| MER | -.067 | 0.26 | -0.27 | 0.057 | -0.06 | 0.05 |
| BxM | -0.13 | -0.04 | -0.17 | -0.04 | -0.14 | -0.05 |
| BxMxP | -0.14 | 0.11 | -0.12 | -0.01 | -0.09 | 0.01 |

Inbreeding coefficient was calculated for each individual using GCTA software program. *F_1_* is calculated based on the variance of the additive genotype; *F_2_* is calculated based on the excess of homozygosity; and *F_3_* is calculated based on the correlation between uniting gametes.

**Table S8 Mean linkage disequilibrium in the five populations at varying map distances.**

|  | r^2^ | | | | | D’ | | | | |
| --- | --- | --- | --- | --- | --- | --- | --- | --- | --- | --- |
|  | BL | PD | MER | BxM | BxMxP | BL | PD | MER | BxM | BxMxP |
| 10Kb | 0.335 | 0.334 | 0.27 | 0.288 | 0.300 | 0.846 | 0.839 | 0.744 | 0.784 | 0.781 |
| 10-20Kb | 0.276 | 0.259 | 0.202 | 0.213 | 0.215 | 0.806 | 0.786 | 0.675 | 0.698 | 0.689 |
| 20-40Kb | 0.223 | 0.206 | 0.141 | 0.152 | 0.156 | 0.754 | 0.728 | 0.573 | 0.603 | 0.597 |
| 40-60Kb | 0.189 | 0.174 | 0.103 | 0.115 | 0.118 | 0.717 | 0.692 | 0.506 | 0.539 | 0.533 |
| 60-100Kb | 0.160 | 0.146 | 0.075 | 0.084 | 0.088 | 0.681 | 0.656 | 0.445 | 0.478 | 0.476 |
| 100-200Kb | 0.133 | 0.120 | 0.049 | 0.058 | 0.063 | 0.644 | 0.620 | 0.382 | 0.418 | 0.420 |
| 200-500Kb | 0.104 | 0.099 | 0.033 | 0.041 | 0.046 | 0.593 | 0.577 | 0.33 | 0.368 | 0.372 |
| 500Kb-1Mb | 0.075 | 0.079 | 0.025 | 0.030 | 0.036 | 0.528 | 0.526 | 0.292 | 0.324 | 0.334 |
| 1-2Mb | 0.047 | 0.058 | 0.018 | 0.021 | 0.027 | 0.456 | 0.467 | 0.254 | 0.279 | 0.295 |
| 2-5Mb | 0.023 | 0.035 | 0.011 | 0.012 | 0.018 | 0.374 | 0.384 | 0.208 | 0.229 | 0.249 |
| 5-10Mb | 0.013 | 0.019 | 0.008 | 0.008 | 0.013 | 0.320 | 0.311 | 0.177 | 0.199 | 0.217 |
| 10-20Mb | 0.009 | 0.011 | 0.006 | 0.007 | 0.010 | 0.293 | 0.261 | 0.159 | 0.184 | 0.195 |
| 20-50Mb | 0.007 | 0.007 | 0.005 | 0.006 | 0.008 | 0.279 | 0.224 | 0.148 | 0.174 | 0.178 |
| 50Mb+ | 0.006 | 0.005 | 0.005 | 0.006 | 0.007 | 0.275 | 0.211 | 0.145 | 0.172 | 0.172 |
| Non-syntenic | 0.009 | 0.011 | 0.006 | 0.007 | 0.009 | 0.292 | 0.244 | 0.174 | 0.183 | 0.188 |

**Table 9 List of the genes located in the region between 49.2 and 51.2 Mb on OAR15.**

| Start | Stop | Symbol | Description | Summary functions in human |
| --- | --- | --- | --- | --- |
| 49249381 | 49249453 | TRNAG-CCC | transfer RNA glycine (anticodon CCC) | |
| 49250115 | 49251119 | LOC101123149 | protein olfactory receptor 52K1-like | Olfactory receptors interact with odorant molecules in the nose, to initiate a neuronal response that triggers the perception of a smell. The olfactory receptor proteins are members of a large family of G-protein-coupled receptors (GPCR) arising from single coding-exon genes. Olfactory receptors share a 7-transmembrane domain structure with many neurotransmitter and hormone receptors and are responsible for the recognition and G protein-mediated transduction of odorant signals. The olfactory receptor gene family is the largest in the genome. The nomenclature assigned to the olfactory receptor genes and proteins for this organism is independent of other organisms. [provided by RefSeq, Jul 2008] |
| 49265666 | 49266685 | LOC101123408 | protein olfactory receptor 52Z1-like | Olfactory receptors interact with odorant molecules in the nose, to initiate a neuronal response that triggers the perception of a smell. The olfactory receptor proteins are members of a large family of G-protein-coupled receptors (GPCR) arising from single coding-exon genes. Olfactory receptors share a 7-transmembrane domain structure with many neurotransmitter and hormone receptors and are responsible for the recognition and G protein-mediated transduction of odorant signals. The olfactory receptor gene family is the largest in the genome. The nomenclature assigned to the olfactory receptor genes and proteins for this organism is independent of other organisms. [provided by RefSeq, Jul 2008] |
| 49270877 | 49310336 | RRM1 | ribonucleotide reductase M1 | This gene encodes one of two non-identical subunits that constitute ribonucleoside-diphosphate reductase, an enzyme essential for the production of deoxyribonucleotides prior to DNA synthesis in S phase of dividing cells. It is one of several genes located in the imprinted gene domain of 11p15.5, an important tumor-suppressor gene region. Alterations in this region have been associated with the Beckwith-Wiedemann syndrome, Wilms tumor, rhabdomyosarcoma, adrenocrotical carcinoma, and lung, ovarian, and breast cancer. This gene may play a role in malignancies and disease that involve this region. [provided by RefSeq, Jul 2008] |
| 49312417 | 49506741 | STIM1 | stromal interaction molecule 1 | This gene encodes a type 1 transmembrane protein that mediates Ca2+ influx after depletion of intracellular Ca2+ stores by gating of store-operated Ca2+ influx channels (SOCs). It is one of several genes located in the imprinted gene domain of 11p15.5, an important tumor-suppressor gene region. Alterations in this region have been associated with the Beckwith-Wiedemann syndrome, Wilms tumor, rhabdomyosarcoma, adrenocrotical carcinoma, and lung, ovarian, and breast cancer. This gene may play a role in malignancies and disease that involve this region, as well as early hematopoiesis, by mediating attachment to stromal cells. Mutations in this gene are associated with fatal classic Kaposi sarcoma, immunodeficiency due to defects in store-operated calcium entry (SOCE) in fibroblasts, ectodermal dysplasia and tubular aggregate myopathy. This gene is oriented in a head-to-tail configuration with the ribonucleotide reductase 1 gene (RRM1), with the 3' end of this gene situated 1.6 kb from the 5' end of the RRM1 gene. Alternative splicing of this gene results in multiple transcript variants. [provided by RefSeq, May 2013] |
| 49527811 | 49529019 | RHOG | ras homolog family member G | This gene encodes a member of the Rho family of small GTPases, which cycle between inactive GDP-bound and active GTP-bound states and function as molecular switches in signal transduction cascades. Rho proteins promote reorganization of the actin cytoskeleton and regulate cell shape, attachment, and motility. The encoded protein facilitates translocation of a functional guanine nucleotide exchange factor (GEF) complex from the cytoplasm to the plasma membrane where ras-related C3 botulinum toxin substrate 1 is activated to promote lamellipodium formation and cell migration. Two related pseudogene have been identified on chromosomes 20 and X. [provided by RefSeq, Aug 2011] |
| 49529678 | 49542947 | PGAP2 | post-GPI attachment to proteins 2 | |
| 49563541 | 49639034 | NUP98 | nucleoporin 98kDa | Signal-mediated nuclear import and export proceed through the nuclear pore complex (NPC), which is comprised of approximately 50 unique proteins collectively known as nucleoporins. The 98 kDa nucleoporin is generated through a biogenesis pathway that involves synthesis and proteolytic cleavage of a 186 kDa precursor protein. This cleavage results in the 98 kDa nucleoporin as well as a 96 kDa nucleoporin, both of which are localized to the nucleoplasmic side of the NPC. Rat studies show that the 98 kDa nucleoporin functions as one of several docking site nucleoporins of transport substrates. The human gene has been shown to fuse to several genes following chromosome translocations in acute myelogenous leukemia (AML) and T-cell acute lymphocytic leukemia (T-ALL). This gene is one of several genes located in the imprinted gene domain of 11p15.5, an important tumor-suppressor gene region. Alterations in this region have been associated with the Beckwith-Wiedemann syndrome, Wilms tumor, rhabdomyosarcoma, adrenocortical carcinoma, and lung, ovarian, and breast cancer. Alternative splicing of this gene results in several transcript variants; however, not all variants have been fully described. [provided by RefSeq, May 2010] |
| 49643777 | 49650973 | CHRNA10 | protein cholinergic receptor nicotinic alpha 10 (neuronal) | |
| 49652293 | 49659760 | ART1 | mRNA ADP-ribosyltransferase 1 | ADP-ribosyltransferase catalyzes the ADP-ribosylation of arginine residues in proteins. Mono-ADP-ribosylation is a posttranslational modification of proteins that is interfered with by a variety of bacterial toxins including cholera, pertussis, and heat-labile enterotoxins of E. coli. The amino acid sequence consists of predominantly hydrophobic N- and C-terminal regions, which is characteristic of glycosylphosphatidylinositol (GPI)-anchored proteins. This gene was previously designated ART2. [provided by RefSeq, Jul 2008] |
| 49668883 | 49671487 | LOC101102334 | protein ecto-ADP-ribosyltransferase 5-like | The protein encoded by this gene belongs to the ARG-specific ADP-ribosyltransferase family. Proteins in this family regulate the function of target proteins by attaching ADP-ribose to specific amino acid residues in their target proteins. The mouse homolog lacks a glycosylphosphatidylinositol-anchor signal sequence and is predicted to be a secretory enzyme. Several transcripts encoding different isoforms have been found for this gene. [provided by RefSeq, Jul 2014] |
| 49675602 | 49679322 | LOC101117960 | mRNA ecto-ADP-ribosyltransferase 5 | |
| 49681065 | 49700334 | LOC101102587 | protein ecto-ADP-ribosyltransferase 5-like | |
| 49714419 | 49717302 | LOC101118216 | protein ecto-ADP-ribosyltransferase 5-like | |
| 49718454 | 49751791 | LOC101102838 | protein short transient receptor potential channel 2-like | |
| 49756097 | 49838480 | RNF121 | mRNA ring finger protein 121 | The protein encoded by this gene contains a RING finger, a motif present in a variety of functionally distinct proteins and known to be involved in protein-protein and protein-DNA interactions. Several alternatively spliced transcript variants have been noted for this gene, however, not all are likely to encode viable protein products. [provided by RefSeq, Sep 2008] |
| 49776258 | 49778818 | LOC101118725 | protein 40S ribosomal protein S3a pseudogene | Ribosomes, the organelles that catalyze protein synthesis, consist of a small 40S subunit and a large 60S subunit. Together these subunits are composed of 4 RNA species and approximately 80 structurally distinct proteins. This gene encodes a ribosomal protein that is a component of the 40S subunit. The protein belongs to the S3AE family of ribosomal proteins. It is located in the cytoplasm. Disruption of the gene encoding rat ribosomal protein S3a, also named v-fos transformation effector protein, in v-fos-transformed rat cells results in reversion of the transformed phenotype. This gene is co-transcribed with the U73A and U73B small nucleolar RNA genes, which are located in its fourth and third introns, respectively. As is typical for genes encoding ribosomal proteins, there are multiple processed pseudogenes of this gene dispersed through the genome. Alternatively spliced transcript variants have been found for this gene. [provided by RefSeq, May 2012] |
| 49840944 | 49843903 | IL18BP | mRNA interleukin 18 binding protein | The protein encoded by this gene functions as an inhibitor of the proinflammatory cytokine, IL18. It binds IL18, prevents the binding of IL18 to its receptor, and thus inhibits IL18-induced IFN-gamma production, resulting in reduced T-helper type 1 immune responses. This protein is constitutively expressed and secreted in mononuclear cells. Elevated level of this protein is detected in the intestinal tissues of patients with Crohn's disease. Alternatively spliced transcript variants encoding different isoforms have been described for this gene. [provided by RefSeq, Feb 2011] |
| 49844600 | 49875612 | NUMA1 | mRNA nuclear mitotic apparatus protein 1 | This gene encodes a large protein that forms a structural component of the nuclear matrix. The encoded protein interacts with microtubules and plays a role in the formation and organization of the mitotic spindle during cell division. Chromosomal translocation of this gene with the RARA (retinoic acid receptor, alpha) gene on chromosome 17 have been detected in patients with acute promyelocytic leukemia. Alternative splicing results in multiple transcript variants. [provided by RefSeq, Nov 2013] |
| 49919686 | 49934136 | LRRC51 | mRNA leucine-rich repeat-containing protein 51 | |
| 49934503 | 49940851 | LAMTOR1 | mRNA late endosomal/lysosomal adaptor MAPK and MTOR activator 1 | |
| 49943502 | 49945846 | TOMT | transmembrane O-methyltransferase | |
| 49946522 | 49948362 | ANAPC15 | mRNA anaphase promoting complex subunit 15 | |
| 49992342 | 49997798 | FOLR3 | mRNA folate receptor 3 (gamma) | This gene encodes a member of the folate receptor (FOLR) family, members of which have a high affinity for folic acid and for several reduced folic acid derivatives, and mediate delivery of 5-methyltetrahydrofolate to the interior of cells. This gene includes two polymorphic variants; the shorter one has two base deletion in the CDS, resulting in a truncated polypeptide, compared to the longer one. Both protein products are constitutively secreted in hematopoietic tissues and are potential serum marker for certain hematopoietic malignancies. The longer protein has a 71% and 79% sequence homology with the FOLR1 and FOLR2 proteins, respectively. [provided by RefSeq, Jul 2008] |
| 50003001 | 50014571 | FOLR1 | mRNA folate receptor 1 (adult) | The protein encoded by this gene is a member of the folate receptor family. Members of this gene family bind folic acid and its reduced derivatives, and transport 5-methyltetrahydrofolate into cells. This gene product is a secreted protein that either anchors to membranes via a glycosyl-phosphatidylinositol linkage or exists in a soluble form. Mutations in this gene have been associated with neurodegeneration due to cerebral folate transport deficiency. Due to the presence of two promoters, multiple transcription start sites, and alternative splicing, multiple transcript variants encoding the same protein have been found for this gene. [provided by RefSeq, Oct 2009] |
| 50018863 | 50018935 | TRNAG-CCC | transfer RNA glycine (anticodon CCC) | |
| 50031556 | 50034889 | FOLR2 | mRNA folate receptor 2 (fetal) | The protein encoded by this gene is a member of the folate receptor (FOLR) family, and these genes exist in a cluster on chromosome 11. Members of this gene family have a high affinity for folic acid and for several reduced folic acid derivatives, and they mediate delivery of 5-methyltetrahydrofolate to the interior of cells. This protein has a 68% and 79% sequence homology with the FOLR1 and FOLR3 proteins, respectively. Although this protein was originally thought to be specific to placenta, it can also exist in other tissues, and it may play a role in the transport of methotrexate in synovial macrophages in rheumatoid arthritis patients. Multiple transcript variants that encode the same protein have been found for this gene. [provided by RefSeq, Jul 2008] |
| 50038091 | 50052170 | INPPL1 | mRNA inositol polyphosphate phosphatase-like 1 | The protein encoded by this gene is an SH2-containing 5'-inositol phosphatase that is involved in the regulation of insulin function. The encoded protein also plays a role in the regulation of epidermal growth factor receptor turnover and actin remodelling. Additionally, this gene supports metastatic growth in breast cancer and is a valuable biomarker for breast cancer. [provided by RefSeq, Jan 2009] |
| 50052618 | 50056577 | PHOX2A | protein paired-like homeobox 2a | The protein encoded by this gene contains a paired-like homeodomain most similar to that of the Drosophila aristaless gene product. The encoded protein plays a central role in development of the autonomic nervous system. It regulates the expression of tyrosine hydroxylase and dopamine beta-hydroxylase, two catecholaminergic biosynthetic enzymes essential for the differentiation and maintenance of the noradrenergic neurotransmitter phenotype. The encoded protein has also been shown to regulate transcription of the alpha3 nicotinic acetylcholine receptor gene. Mutations in this gene have been associated with autosomal recessive congenital fibrosis of the extraocular muscles. [provided by RefSeq, Jul 2008] |
| 50087186 | 50226492 | CLPB | mRNA ClpB homolog mitochondrial AAA ATPase chaperonin | This gene belongs to the ATP-ases associated with diverse cellular activities (AAA+) superfamily. Members of this superfamily form ring-shaped homo-hexamers and have highly conserved ATPase domains that are involved in various processes including DNA replication, protein degradation and reactivation of misfolded proteins. All members of this family hydrolyze ATP through their AAA+ domains and use the energy generated through ATP hydrolysis to exert mechanical force on their substrates. In addition to an AAA+ domain, the protein encoded by this gene contains a C-terminal D2 domain, which is characteristic of the AAA+ subfamily of Caseinolytic peptidases to which this protein belongs. It cooperates with Hsp70 in the disaggregation of protein aggregates. Allelic variants of this gene are associated with 3-methylglutaconic aciduria, which causes cataracts and neutropenia. Alternative splicing results in multiple transcript variants. [provided by RefSeq, Apr 2015] |
| 50337866 | 50432640 | PDE2A | mRNA phosphodiesterase 2A cGMP-stimulated | |
| 50442605 | 50478503 | ARAP1 | mRNA ArfGAP with RhoGAP domain ankyrin repeat and PH domain 1 | The protein encoded by this gene contains SAM, ARF-GAP, RHO-GAP, ankyrin repeat, RAS-associating, and pleckstrin homology (PH) domains. In vitro, this protein displays RHO-GAP and phosphatidylinositol (3,4,5) trisphosphate (PIP3)-dependent ARF-GAP activity. The encoded protein associates with the Golgi, and the ARF-GAP activity mediates changes in the Golgi and the formation of filopodia. It is thought to regulate the cell-specific trafficking of a receptor protein involved in apoptosis. Multiple transcript variants encoding different isoforms have been found for this gene. [provided by RefSeq, Sep 2008] |
| 50501838 | 50536774 | STARD10 | protein StAR-related lipid transfer (START) domain containing 10 | |
| 50553550 | 50569219 | ATG16L2 | mRNA autophagy related 16-like 2 (S. cerevisiae) | |
| 50576386 | 50817451 | FCHSD2 | mRNA FCH and double SH3 domains 2 | |
| 50964501 | 50988822 | P2RY6 | mRNA pyrimidinergic receptor P2Y G-protein coupled 6 | The product of this gene belongs to the family of P2 receptors, which is activated by extracellular nucleotides and subdivided into P2X ligand-gated ion channels and P2Y G-protein coupled receptors. This family has several receptor subtypes with different pharmacological selectivity, which overlaps in some cases, for various adenosine and uridine nucleotides. This receptor, which is a G-protein coupled receptor, is responsive to UDP, partially responsive to UTP and ADP, and not responsive to ATP. It is proposed that this receptor mediates inflammatory responses. Alternative splicing results in multiple transcript variants that encode different protein isoforms. [provided by RefSeq, Mar 2013] |
| 50996826 | 51052781 | ARHGEF17 | mRNA Rho guanine nucleotide exchange factor (GEF) 17 | |
| 51059947 | 51077198 | RELT | protein RELT tumor necrosis factor receptor | The protein encoded by this gene is a member of the TNF-receptor superfamily. This receptor is especially abundant in hematologic tissues. It has been shown to activate the NF-kappaB pathway and selectively bind TNF receptor-associated factor 1 (TRAF1). This receptor is capable of stimulating T-cell proliferation in the presence of CD3 signaling, which suggests its regulatory role in immune response. Two alternatively spliced transcript variants of this gene encoding the same protein have been reported. [provided by RefSeq, Jul 2008] |
| 51085992 | 51285804 | FAM168A | mRNA family with sequence similarity 168 member A | |
