# Supplementary material for: Genome-wide linkage disequilibrium and genetic diversity in five populations of Australian domestic sheep
Source: Genet Sel Evol. 2015 Nov 24;47:90. doi: 10.1186/s12711-015-0169-6 (PMC4659207; doi:10.1186/s12711-015-0169-6)
Supplement: Supplementary file 2 — 10.1186/s12711-015-0169-6 Distribution of minor allele frequency (MAF) for each population studied. The percentage of SNP is plotted for each frequency bin. Figure S2. Average D' values for each population. [file 12711_2015_169_MOESM2_ESM.docx]

**Figure S1** Distribution of minor allele frequency (MAF) for each population studied. The percentage of SNP is plotted for each frequency bin.

**Figure S2** Average D' values for each population.
